# Antioxidant Capacity and Polyphenolic Profile of Extractable and Non-Extractable Fractions of Traditional Mediterranean Diet Recipes from Different Regions

**DOI:** 10.3390/antiox15030377

**Published:** 2026-03-18

**Authors:** Marta Cuenca-Ortola, Mónica Gandía, Salah Chaji, Fatima Zahrae El Mossaid, Said Ennahli, El Amine Ajal, Stefania Filice, Achraf Ammar, Amparo Gamero, Antonio Cilla

**Affiliations:** 1 Nutrition and Food Science Area, Faculty of Pharmacy and Food Sciences, University of Valencia, Burjassot, 46100 Valencia, Spain; marta.cuenca@uv.es (M.C.-O.); antonio.cilla@uv.es (A.C.); 2 Food Technology Area, Faculty of Pharmacy and Food Sciences, University of Valencia, Burjassot, 46100 Valencia, Spain; monica.gandia@uv.es; 3 Agro-pôle Olivier, National School of Agriculture, Meknès 50001, Morocco; salahchaji1@gmail.com (S.C.); fatimazahrae.elmossaid9@gmail.com (F.Z.E.M.); ennahlisaid@gmail.com (S.E.); 4 UPR of Pharmacognosy, Faculty of Medicine and Pharmacy of Rabat, Mohammed V University, Rabat 6203, Morocco; 5 Microtarians SIS, Société d’Impact Societal, Luxembourg 1746, Luxembourg; stefania.filice@microtarians.com; 6 Department of Training and Movement Science, Institute of Sport Science, Johannes Gutenberg University Mainz, 55128 Mainz, Germany; acammar@uni-mainz.de; 7 Research Laboratory, Molecular Bases of Human Pathology, Faculty of Medicine, University of Sfax, Sfax 30000, Tunisia

**Keywords:** ORAC, TEAC, polyphenols, antioxidant capacity, Mediterranean recipes, HPLC-DAD, nutritional profile

## Abstract

The Mediterranean Diet (MD) is recognized for its nutritional quality, health-promoting properties, and richness in bioactive compounds, yet studies analyzing complete traditional recipes considering both extractable and non-extractable fractions are limited. This study characterized the total antioxidant capacity (TAC) and phenolic profile of 56 traditional MD recipes from eight countries, grouped into European Mediterranean (France, Italy, and Spain), African Mediterranean (Tunisia, Algeria, and Morocco), and non-Mediterranean European (Luxembourg and Germany) regions. Samples were freeze-dried and subjected to aqueous-organic extraction followed by acid hydrolysis. TAC was measured using TEAC, ORAC, and total phenolics (Folin–Ciocalteu, reflecting reducing capacity), while phenolic profiles were analyzed by HPLC-DAD. Relationships between phenolics and TAC were evaluated using linear and mixed-effects models, accounting for country-level heterogeneity. Mediterranean recipes showed higher TAC and greater phenolic diversity than non-Mediterranean recipes, with a predominance of phenolic acids, secoiridoids, and flavonoids, reflecting characteristic olive oil use. In all regions, the non-extractable fraction contributed >80% to TAC, highlighting underestimation by conventional methods and its dominant contribution to dietary antioxidant intake. TEAC was positively associated with extractable phenolics, whereas ORAC reflected country-specific culinary features independently of total phenolic content. These findings underscore the significant bioactive potential of traditional MD recipes, which can be considered functional foods, and the importance of comprehensive evaluations of both extractable and non-extractable fractions for nutritional research and dietary interventions.

## 1. Introduction

The Mediterranean Diet (MD) is distinguished by its abundance in antioxidant compounds, such as vitamins A, C, and E, minerals such as selenium (Se) and zinc (Zn), plant sterols, phenolic and organosulfur compounds, and carotenoids, which are essential for combating free radicals responsible for oxidative stress [1,2]. In this regard, several studies have demonstrated a positive relationship between higher antioxidant consumption and a lower predisposition to cardiovascular diseases, neurodegenerative diseases, diabetes, and more [3,4,5,6,7,8,9]. These antioxidants are primarily found in plant-based foods, such as fruits and vegetables [10].

The health-promoting properties of the MD, which is predominantly plant-based, are mainly linked to its richness in antioxidant compounds [11]. Indeed, besides the abundant use of grains, nuts, beans, fruits, and vegetables, olive oil, the emblematic ingredient of the MD, stands as a fundamental component and a principal source of fat [2,12,13]. Although oleic acid, its predominant fatty acid, is considered to be its most prominent bioactive constituent, olive oil health effects depend also on, or are potentiated scientifically, by other minor components, mainly polyphenols [14,15,16]. Owing to their potent antioxidant activity, these metabolites are believed to play a crucial role in conferring a wide range of health benefits within olive oil-based dietary habits [17,18]

Polyphenols are a large group of secondary metabolites that possess one or more aromatic rings attached to one or multiple hydroxyl groups. The most commonly found polyphenols in Mediterranean food ingredients include phenolic acids, flavonoids, stilbenes, lignans, and secoiridoids [19,20]. Mediterranean recipes exhibit various and complex phenolic profiles that arise from both the raw ingredients (e.g., olive oil, tomatoes, herbs, vegetables) and the culinary processes used in their preparation. For instance, olive oil contributes mostly simple phenols and secoiridoids, notably hydroxytyrosol, tyrosol, and oleuropein derivatives, whose contents depend on cultivar, geographical origin, oil-extraction method, and storage conditions [20,21]. These compounds not only enrich flavor but also enhance antioxidant potential when incorporated into Mediterranean recipes [22,23]. Likewise, aromatic herbs (e.g., oregano, thyme, rosemary, and sage), which are widely used across Mediterranean cuisine, are also rich sources of phenolic acids, flavones, and terpenoid phenolics [24,25]. However, both ingredient choice and culinary practices substantially modify the phenolic content and antioxidant activity of the recipes. In a stimulant example, recent studies highlighted that cooking technique (i.e., method, time, and temperature) can exert a greater effect on the final phenolic composition than simple thermal exposure, highlighting the need to consider culinary practices when preparing MD dishes [24,25,26]. A study on *sofrito* formulations also showed that ingredient proportions and thermal treatment remarkably altered total polyphenols, specific phenolic acids, and flavonoids [27].

Nevertheless, few studies have evaluated the total antioxidant capacity (TAC) and total polyphenol (TP) content in complete recipes [6,7,28,29,30,31] (as foods are usually ingested), although there is considerable information available in the literature regarding individual ingredients. Generally, the interactions between food nutrients, known as “food synergy,” are not considered. Additionally, nutritional and antioxidant properties can vary depending on the technological treatments applied during preparation [32].

The structural diversity of foods, coupled with potential interactions between them, various mechanisms of action, and their biological roles, complicates the evaluation of a reliable procedure to measure antioxidant capacity [33]. In this study, TAC was evaluated using TEAC (electron transfer-based (ET)) and ORAC (hydrogen atom transfer-based (HAT)) radical-scavenging assays, complemented by TP (Folin–Ciocalteu method) to assess total reducing capacity [34]. To carry out this evaluation, three key steps must be considered: the extraction of compounds, the measurement of antioxidant capacity, and the interpretation of results [35,36]. Extraction is a crucial process. So far, different studies have used various organic solvents, such as ethanol, methanol, and acetone, or combinations of these. However, it has been shown that an acidic mixture of methanol and water provides the highest capacity for extracting antioxidant compounds [37]. Additionally, there are studies that have employed other techniques, such as alkaline hydrolysis, acid hydrolysis, or enzymatic digestion [38].

It is important to differentiate and analyze extractable antioxidants (obtained with aqueous-organic solvents) and non-extractable antioxidants (released from the residue through acid hydrolysis) because both contribute significantly to the TAC of foods [1,39]. Non-extractable antioxidants represent phenolic compounds and other antioxidants that remain bound or trapped within the food matrix. This fraction is often overlooked in conventional analyses, yet it can contribute between 30% and 80% of the total antioxidant content, especially in fiber-rich foods such as cereals, legumes, and fruits. The inclusion of data on both types in databases such as EBASIS [40], along with quality evaluation and the analytical methods used, enables a more complete and accurate understanding of their contribution to the overall antioxidant activity of foods, underscoring the importance of their study.

The presence of these antioxidants in cooked, complete recipes has been studied [28,29,30], emphasizing the importance of analyzing the non-extractable fraction, as it significantly contributes to total intake of antioxidants (around 60% to 80% of the total). Most studies available in literature report only the daily intake of the extractable fraction. Studies such as those by Arranz Martínez et al. [41] estimated both fractions in a complete Spanish diet and their intestinal bioavailability. According to those authors, the amount of non-extractable antioxidants was almost double that of extractable. Likewise, other authors have determined TAC and TP to measure both fractions of antioxidants in dishes that are not subjected to aggressive cooking techniques, showing higher values in non-extractable fractions [28,29,30,31,42]. Therefore, the analysis of total antioxidant properties in whole dishes and recipes may help evaluate interactions within the food matrix [43]. A greater understanding of antioxidants and their distribution in foods contributes to a better understanding of their impact on health, thereby facilitating the adoption of healthier eating habits, and may contribute to a better understanding of their potential role in the prevention of chronic degenerative diseases.

In this context, the objective of this study is to provide novel information on the TAC (TEAC, ORAC) and TP of 56 traditional MD recipes from eight different countries, considering both extractable and non-extractable fractions and providing, for the first time, their polyphenolic profile. Thus, this research can provide antioxidant properties of foods as a whole dish and facilitate further nutrition and clinical-related studies encouraging the consumption of MD recipes high in antioxidants from different regions [44].

## 2. Materials and Methods

### 2.1. Recipes: Identification of Recipes, Sampling and Dish Preparation

A total of 56 recipes were gathered from 8 different countries, with 48 from Mediterranean countries and 8 from non-Mediterranean countries. In the Supplementary Material, the recipes with their ingredients, nutritional information, and the procedure for cooking them can be found. Initially, each nation contributed between 10 and 14 traditional and representative recipes. Later, the Microtarians group, an educational center from Luxembourg, applied several selection criteria: (i) authenticity of the recipe, assessing whether it was truly traditional or had been modified over time, prioritizing dishes deeply rooted in each country’s culinary tradition; (ii) ease of preparation, aiming to make the recipes accessible to people with varying levels of cooking skills; (iii) cultural relevance, ensuring that each selected recipe reflected the cultural identity and diversity of its country of origin; (iv) variety, seeking to include dishes that showcased the richness of flavors and ingredients typical of the national cuisine; (v) packaging feasibility, considering the practicality of transporting and distributing the recipes; and (vi) potential for enhancement in terms of antioxidant content and nutritional value, with the goal of selecting recipes that could be improved without compromising their authenticity. The final number of recipes per country was determined by design to ensure representativeness: 8 recipes were selected for each of the six Mediterranean countries (France, Italy, Spain, Algeria, Morocco, and Tunisia), while 4 recipes were selected for each of the two non-Mediterranean countries (Luxembourg and Germany), reflecting their partial adherence to the Mediterranean dietary pattern.

A total of 56 recipes were gathered from 8 different countries, with 48 from Mediterranean countries and 8 from non-Mediterranean countries. In the Supplementary Material, the recipes with their ingredients, nutritional information, and the procedure for cooking them can be found. Initially, each nation contributed between 10 and 14 traditional and representative recipes. Later, the Microtarians group, an educational center from Luxembourg, applied several selection criteria: (i) authenticity of the recipe, assessing whether it was truly traditional or had been modified over time, prioritizing dishes deeply rooted in each country’s culinary tradition; (ii) ease of preparation, aiming to make the recipes accessible to people with varying levels of cooking skills; (iii) cultural relevance, ensuring that each selected recipe reflected the cultural identity and diversity of its country of origin; (iv) variety, seeking to include dishes that showcased the richness of flavors and ingredients typical of the national cuisine; (v) packaging feasibility, considering the practicality of transporting and distributing the recipes; and (vi) potential for enhancement in terms of antioxidant content and nutritional value, with the goal of selecting recipes that could be improved without compromising their authenticity. The final number of recipes per country was determined by design to ensure representativeness: 8 recipes were selected for each of the six Mediterranean countries (France, Italy, Spain, Algeria, Morocco, and Tunisia), while 4 recipes were selected for each of the two non-Mediterranean countries (Luxembourg and Germany), reflecting their partial adherence to the Mediterranean dietary pattern.

A total of 56 recipes were gathered from 8 different countries, with 48 from Mediterranean countries and 8 from non-Mediterranean countries. In the Supplementary Material , the recipes with their ingredients, nutritional information, and the procedure for cooking them can be found. Initially, each nation contributed between 10 and 14 traditional and representative recipes. Later, the Microtarians group, an educational center from Luxembourg, applied several selection criteria: (i) authenticity of the recipe, assessing whether it was truly traditional or had been modified over time, prioritizing dishes deeply rooted in each country’s culinary tradition; (ii) ease of preparation, aiming to make the recipes accessible to people with varying levels of cooking skills; (iii) cultural relevance, ensuring that each selected recipe reflected the cultural identity and diversity of its country of origin; (iv) variety, seeking to include dishes that showcased the richness of flavors and ingredients typical of the national cuisine; (v) packaging feasibility, considering the practicality of transporting and distributing the recipes; and (vi) potential for enhancement in terms of antioxidant content and nutritional value, with the goal of selecting recipes that could be improved without compromising their authenticity. The final number of recipes per country was determined by design to ensure representativeness: 8 recipes were selected for each of the six Mediterranean countries (France, Italy, Spain, Algeria, Morocco, and Tunisia), while 4 recipes were selected for each of the two non-Mediterranean countries (Luxembourg and Germany), reflecting their partial adherence to the Mediterranean dietary pattern.

### 2.2. Sampling

The samples were cooked by the Mediterráneo Culinary Center (MCC) (Valencia), (https://mediterraneoculinary.com/) following the elaboration procedure indicated in the Supplementary Material. The ingredients were purchased in bulk from local suppliers using standard national commercial brands, ensuring consistency in quality and origin across all preparations. They were homogenized at maximum speed using a household chopper and weighed on a precision balance ± 0.001 g (Kern PNS, Regensburg, Germany). Subsequently, they were placed in containers (Tupperware-Hacendado; Madrid, Spain) and freeze-dried in the Spanish National Research Council—Institute of Agrochemistry and Food Technology (CSIC-IATA) (freeze-dried Telstar, LyoBeta) over approximately 72 h, with an initial condenser temperature of −80 °C, a vacuum of 600 µbar, and a mean sample temperature of −80 °C, reaching final conditions of approximately −80 °C, 21 µbar, and 28 °C, respectively. Once freeze-dried, samples were vacuum-packed at maximum vacuum until analysis.

### 2.3. Antioxidant Extraction Procedure

Freeze-dried and ground samples were used for antioxidant extraction. Extractable (aqueous-organic extracts) and non-extractable (acid hydrolysis) antioxidants were extracted as described by [28,29,30].

#### 2.3.1. Aqueous-Organic Extract

For the extraction of extractable antioxidants in the aqueous-organic extract, the samples must first undergo a process of crushing, freeze drying, and grinding to ensure proper homogenization. Subsequently, between 3 and 5.5 g of the sample were weighed into glass tubes, to which 20 mL of a methanol: water (50:50, *v*/*v*) solution acidified with 2N HCl was added until a pH of 2.0 was reached. The mixture was stirred at room temperature using a vortex and a universal platform shaker (IKA, KS260) for 1 h at a speed of 100 rpm, promoting the extraction of the target compounds.

After agitation, the suspension was centrifuged (centrifuge S810R, Eppendorf, Hamburgo, Germany ) at 978× *g* for 10 min. The resulting supernatant was carefully recovered using a glass Pasteur pipette and transferred to a 50 mL Pyrex tube.

To maximize antioxidant extraction, 20 mL of an acetone:water (70:30, *v*/*v*) mixture was added to the remaining solid residue, and the agitation and centrifugation processes were repeated once under the same conditions. The supernatants obtained from both extractions were combined and subjected to a final centrifugation at 1227× *g* for 15 min. Finally, the resulting supernatant was collected, and the total volume was adjusted to 50 mL, yielding the final extract ready for analysis.

#### 2.3.2. Acid Hydrolysis Residue

The residual solids were dried at 25 °C in an oven (ule 500 ao, Memmert, Schwabach, Germany) overnight. Samples were weighed before and after drying to ensure a constant weight, with variations not exceeding 0.05 g. Once dried, 20 mg of the residue was mixed with 2 mL of methanol and 0.2 mL of concentrated sulfuric acid (18 M). The mixture was vortexed for 1 min and subsequently heated in a thermoblock (Stuart block heater SBH200D, Buch & Holm, Herlev, Denmark) at 85 °C for 20 h, with intermittent stirring every 1–2 h.

Following the heating process, the sample was centrifuged at 3000× *g* for 10 min. The resulting supernatant was carefully collected and transferred to a clean 10 mL Pyrex glass tube. The remaining residue was then subjected to two washing steps using minimal volumes of approximately 1 mL of Milli-Q water. Each wash was followed by centrifugation at 3000× *g* for 10 min. The supernatants from both washes were combined with the initial extract, and the final volume was adjusted to 5 mL with Milli-Q water.

### 2.4. Determination of Antioxidant Capacity and Total Polyphenols

#### 2.4.1. TEAC Determination

The determination of antioxidant capacity using the TEAC method was conducted following the procedure described by Re, et al. [45]. This essay evaluates the ability to scavenge free radicals present in the medium. The greenish blue ABTS+ cation radical is generated through the reaction between 2,2′-azinobis (3-ethylbenzothiazoline-6-sulfonic acid) (ABTS) and potassium persulfate (K_2_S_2_O_8_). This radical is a stable, water-soluble compound characterized by a distinct absorption spectrum. Upon adding the samples to the radical solution, a decrease in absorbance occurs, allowing the antioxidant capacity to be determined based on the sample’s ability to reduce the radical concentration. The results are expressed relative to 6-hydroxy-2,5,7,8-tetramethylchroman-2-carboxylic acid (Trolox).

The ABTS+ stock solution was prepared by dissolving 7 mM ABTS and 140 mM K_2_S_2_O_8_ and allowing the mixture to react in darkness at room temperature for 12–16 h, ensuring stability for up to two days. Before analysis, the ABTS solution was diluted approximately 1/100 with ethanol and adjusted to an absorbance of 0.70 ± 0.02 at 734 nm at 30 °C using a thermostat-controlled spectrophotometer (PerkinElmer UV-VIS Lambda 365—PerkinElmer; Waltham, MA, USA). A 5 mM Trolox stock solution was prepared in volumetric flasks.

For the analysis, 2 mL of the ABTS radical solution was first added to the cuvette to measure its initial absorbance, followed by the addition of 100 µL of the sample or standard solution. After 3 min, the absorbance was measured again. The analysis was performed in triplicate, and the antioxidant capacity was expressed as Trolox equivalents (TE) in µM per 100 g of dry weight (DW). The dilution of each extract—both extractable and non-extractable fractions—was previously optimized to achieve an inhibition percentage within the recommended 20–80% range for the TEAC assay. Dilutions were carried out using ethanol as the solvent, with specific dilution factors ranging from 1:1 to 1:3 *v*/*v* depending on the sample.

#### 2.4.2. ORAC Determination

The ORAC method was developed based on the ability of antioxidant compounds to capture oxygen radicals [46]. This ability was assessed by measuring the reduction in fluorescence of fluorescein, caused by oxidative damage from free radicals generated through the thermal decomposition of 2,2-azinobis(2-amidinopropane) dihydrochloride (AAPH). The antioxidant capacity of the sample was determined by evaluating the time and percentage of protection against fluorescence loss, using Trolox as the standard antioxidant. The reaction took place using a Multilabel Plate Counter VICTOR^3^ 1420 (PerkinElmer; Waltham, MA, USA), equipped with a fluorescence filter for excitation at 485 nm and emission at 535 nm. The assays were conducted at 37 °C, with sodium fluorescein and AAPH at concentrations of 0.015 and 120 mg/mL, respectively. The samples were diluted in a 75 mM phosphate buffer, pH 7.4. The dilution ratios of the samples ranged from 1:50 to 1:250 *v*/*v*, with most samples diluted at 1:50 *v*/*v*. In a white multi-well plate, 80 µL of fluorescein, 40 µL of AAPH, and 80 µL of the diluted sample, Trolox (standard), or phosphate buffer (blank) were added. Fluorescence was measured every 5 min for 90 min until it dropped below 5% of the initial value. The results were expressed as TE µM/100 g DW. The analysis was performed in quadruplicate.

#### 2.4.3. TP Determination

To determine TP, the Folin–Ciocalteu reagent was used, which is prepared by combining phosphotungstic acid and phosphomolybdic acid. In the presence of an excess of this reagent, polyphenols reduce the acids to blue tungsten and molybdenum oxides. When the sample was treated with the reagent in the presence of sodium carbonate (Na_2_CO_3_), the absorbance was directly proportional to the polyphenol concentration [41]. The method included centrifuging the non-extractable polyphenol samples at 3000× *g* for 10 min to remove any suspended particles formed during freezing. Then, 100 µL of the sample (whether extractable or non-extractable antioxidants) was mixed with 3 mL of a 2% (*w*/*v*) sodium carbonate solution and 100 µL of a 50% (*w*/*v*) Folin–Ciocalteu reagent. The mixture was allowed to react for one hour at room temperature in the dark, after which the absorbance was measured at 765 nm using a spectrophotometer (Lambda 365 UV-VIS, Perkin Elmer, Shelton, CT, USA) ). Quantification was carried out using a calibration curve based on gallic acid standards within a range of 0–300 mg/L. The results were expressed as gallic acid equivalents (GAE) per 100 g DW to allow for comparison with existing literature. Each sample was analyzed in triplicate.

### 2.5. Polyphenol Profile

The phenolic fraction was obtained according to a previously reported method based on an ultrasound-assisted solid–liquid extraction protocol [47]. Briefly, 100 mg of each sample was extracted sequentially using ethanol-water mixtures of increasing ethanol content (60:40 *v*/*v* for the first step and 80:20 *v*/*v* for the second), followed by a final extraction step with pure ethanol. Each step involved 30 min of ultrasound-assisted extraction, centrifugation at 8603× *g* for 10 min and collection of the upper phase. Finally, the resulting supernatants were combined, and 1 mL aliquots were filtered through 0.22 μm Clarinert^®^ nylon syringe filters (Agela Technologies, Torrance, CA, USA) before being transferred into amber glass vials for HPLC analysis. Furthermore, composite samples were prepared for each country by pooling and homogenizing 500 mg from each recipe. The resulting samples were analyzed for their phenolic composition using the same extraction and chromatographic analysis protocol as for the individual recipes to obtain an integrative phenolic profile representative of each country.

Chromatographic analyses were performed on a liquid chromatography system (Agilent 1260 Infinity II LC System, Agilent, Santa Clara, CA, USA) equipped with a diode array detector (DAD, model G7115A). Metabolite separation was achieved on a Zorbax Extend-C18 column (100 × 4.6 mm, 1.8 μm particle size; Agilent Technologies) maintained at 40 °C, with an injection volume of 10 μL. The mobile phase consisted of water (phase A) and acetonitrile (phase B), both acidified with 1% acetic acid. Elution was carried out using a gradient program at a constant flow rate of 1 mL/min as follows: 0–10 min, 10–25% B; 10–12 min, 25–60% B; 12–14 min, 60–80% B; 14–18 min, 80–100% B (held for 2 min); and 20–21 min, 100–10% B, followed by a 3 min equilibration period before the next run. The column oven was maintained at 40 °C, and detection was performed at optimal wavelengths of 240 nm, 280 nm and 330 nm using the DAD detector. External calibration curves (0.1–500 mg/ L) were prepared in ethanol-water (80:20, *v*/*v*) using analytical-grade standards for the quantification of all target analytes. Stock solutions and sample extracts were kept at −32 °C to ensure compound stability prior to analysis. All HPLC-grade standards were purchased from Merck (Darmstadt, Germany) and Sigma-Aldrich (St. Louis, MO, USA). The standards employed in this study comprised the following phenolic and related compounds: apigenin, apigenin-7-*O*-glucoside, caffeic acid, catechin, chlorogenic acid, citric acid, cyanidin 3-*O*-glucoside chloride, cyanidin 3-rutinoside chloride, delphinidin 3-rutinoside chloride, epicatechin, ferulic acid, gallic acid, hydroxytyrosol, isorhamnetin, kaempferol, luteolin, myricetin, naringenin, oleacein, oleocanthal, oleuropein, pelargonidin 3-glucoside chloride, pinoresinol, p-coumaric acid, protocatechuic acid, procyanidin B1, quercetin, quercetin-3-glucoside, resveratrol, rosmarinic acid, rutin, sinapic acid, syringic acid, tyrosol, vanillic acid, and verbascoside.

To enhance the reliability of compound identification, a complementary analysis using liquid chromatography coupled with mass spectrometry (LC-MS) was performed on selected extracts following the HPLC-DAD analysis. Specifically, pooled extracts and a composite sample prepared by mixing equal volumes of all individual extracts were analyzed to confirm the identity of the phenolic compounds detected during chromatographic profiling. LC–MS measurements were conducted using an Agilent 1200 Series high-performance liquid chromatography system (Agilent Technologies, Santa Clara, CA, USA) equipped with a binary pump, online degasser, autosampler, column thermostat, and diode array detector and controlled by OpenLab ChemStation B.04.03 software (Agilent Technologies, Santa Clara, CA, USA). The chromatographic system was coupled to a micrOTOF-Q II mass spectrometer (Bruker Daltonik, Bremen, Germany) with a quadrupole time-of-flight (Q-TOF) analyzer and an electrospray ionization (ESI) source. Mass spectrometer calibration was performed externally before the analytical runs using a sodium formate cluster solution delivered by a syringe pump (Cole-Parmer 74900-00-05, Vernon Hills, IL, USA) equipped with a Hamilton syringe (Reno, NV, USA). The calibration mixture consisted of 5 mM sodium hydroxide in a sheath liquid of 0.2% formic acid in water/isopropanol (1:1, *v*/*v*). This calibration solution was introduced at the start of each run, and the resulting spectra were calibrated before compound identification. The eluent from the LC column was directed to the mass spectrometer through a 1:4 flow splitter. Ionization was performed in electrospray mode under the following conditions: capillary voltage 3200 V, drying gas (nitrogen) flow rate 9 L min^−1^, drying gas temperature 300 °C, and nebulizer pressure 30 psi. Ion Charge Control (ICC) was set to 10,000, and mass spectra were acquired over an *m*/*z* range of 50–1000. Instrument control and data processing were performed using Esquire Control and Data Analysis 4.0 software (Bruker Daltonics, Bremen, Germany).

The LC-MS analysis confirmed the identities of phenolic compounds based on their accurate mass measurements and fragmentation patterns, thereby supporting the peak assignments used for quantitative HPLC-DAD analysis.

### 2.6. Construction of the Data Matrix and Standardization of Variables for Statistical Modeling

Total polyphenol content (TP_total) was defined as the sum of extractable (TP_ext) and non-extractable (TP_non_ext) polyphenols, quantified using established analytical procedures. Extractable polyphenols represent compounds released under conventional solvent extraction, whereas non-extractable polyphenols comprise polyphenolic compounds bound to the food matrix and recovered following additional hydrolysis steps.

In addition to these quantitative measures, major phenolic families (e.g., flavonoids and phenolic acids) were quantified at the individual recipe level using HPLC-DAD analysis. Composite samples prepared at the country level were used only for descriptive characterization of phenolic profiles and were not included in the regression analyses. Accordingly, the statistical unit of analysis for all regression models corresponded to individual recipes (N = 56).

Phenolic-family concentrations were calculated as the sum of individually quantified compounds within each phenolic class, expressed in mg/kg dry weight. Each compound was quantified using its corresponding analytical calibration standard. Because these values represent aggregated concentrations derived from multiple standards and analytical responses, phenolic-family variables were s treated as semi-quantitative enrichment indicators rather than compositional fractions of total polyphenols.

For regression analyses, phenolic-family variables were standardized across recipes using z-score transformation:

z = (x − μ)/σ

where x represents the recipe-level concentration, μ the sample mean, and σ the standard deviation. Standardization was applied to place variables measured on different analytical scales on a comparable metric and to facilitate interpretation of regression coefficients.

Standardized phenolic-family variables were included as exploratory predictors to evaluate potential associations with antioxidant capacity beyond extractable total polyphenol content and were not interpreted as proportional contributions to total polyphenols.

The analytical framework followed a structured modeling strategy designed to address distinct, conceptually driven research questions, progressing from overall polyphenol quantity (dose-response) to fractionation (extractable vs. non-extractable) and to exploratory assessment of phenolic subclass enrichment. Antioxidant capacity was evaluated separately using TEAC and ORAC assays due to their differing chemical principles and sensitivities. To assess the robustness of associations and account for potential non-independence of recipes originating from the same country, mixed-effects modeling was additionally applied to explore between-country heterogeneity.

### 2.7. Statistical Analysis

The results were presented as mean ± standard deviation. An analysis of variance (ANOVA), followed by Tukey’s multiple range post hoc test, was performed to compare the different variables (extractable, non-extractable, and TP, as well as TAC) within each group of samples from the same country. A significant level of *p* < 0.05 was considered for all statistical tests. All analyses were carried out using GraphPad Prism version 8 software.

All regression analyses were conducted using IBM SPSS Statistics (version 29.0; IBM Corp., Armonk, NY, USA), with statistical significance set a priori at *p* < 0.05. Associations between polyphenol variables and antioxidant capacity were examined using linear regression models fitted separately for TEAC and ORAC. Results are reported as unstandardized regression coefficients (b) with standard errors, 95% confidence intervals, t-values, and *p*-values, while model fit was assessed using the coefficient of determination (R^2^) and adjusted R^2^. Ordinary one-way ANOVA assuming Gaussian distribution of residuals was performed, with homoscedasticity formally assessed using Brown–Forsythe’s and Bartlett’s tests. No data transformations were applied and no outliers were excluded.

All regression analyses were conducted using values expressed on a dry-weight (DW) basis to ensure consistency across antioxidant and polyphenol variables and to minimize variability related to differences in moisture content between recipes.

Models were implemented sequentially to reflect the predefined analytical framework, including dose–response models based on total polyphenol content, multiple regression models incorporating extractable polyphenols and exploratory phenolic family indicators, and fraction models jointly including extractable and non-extractable polyphenols.

For all linear regression models, assumptions of linearity, homoscedasticity, normality of residuals, and absence of multicollinearity were evaluated using graphical and statistical diagnostics. Linearity and homoscedasticity were assessed through inspection of residual-versus-fitted plots and standardized residual plots. Normality was evaluated on model residuals rather than raw variables, using histograms and normal probability (Q–Q) plots. Residual distributions showed no substantial deviations from normality, and therefore no data transformations were applied.

Potential outliers and influential observations were examined using standardized residuals, Cook’s distance, and leverage statistics. No observations exceeded commonly accepted thresholds (|standardized residual| > 3 or Cook’s distance > 1), and all observations were retained in the analyses. Multicollinearity was evaluated using variance inflation factors (VIF) and tolerance statistics, with VIF values < 5 considered indicative of acceptable collinearity.

To account for potential clustering of recipes within countries and to explore between-country heterogeneity, linear mixed-effects models with a random intercept for country were conducted as sensitivity analyses. Country was included as a random factor to account for the hierarchical structure of the dataset, in which recipes originating from the same country may share culinary practices, ingredient combinations, and preparation methods that could lead to statistical non-independence of observations. The mixed-effects models were therefore intended to control for potential within-country correlation and to evaluate the robustness of fixed-effect associations rather than to support population-level inference across countries. Mixed-effects models mirrored the fixed-effects structure of the corresponding linear regression models and were performed separately for TEAC and ORAC. Fixed-effect estimates are reported as unstandardized coefficients with 95% confidence intervals and *p*-values derived from Type III tests.

Model performance was evaluated using restricted log-likelihood (−2LL) and Akaike’s Information Criterion (AIC). Variance partitioning was assessed using intraclass correlation coefficients (ICC), and pseudo-R^2^ values were reported as marginal R^2^ (variance explained by fixed effects) and conditional R^2^ (variance explained by both fixed and random effects).

Given the modest sample size and limited number of observations per country, regression models were specified in a parsimonious manner, and mixed-effects analyses were interpreted cautiously as sensitivity analyses.

## 3. Results

**Figure 1:**
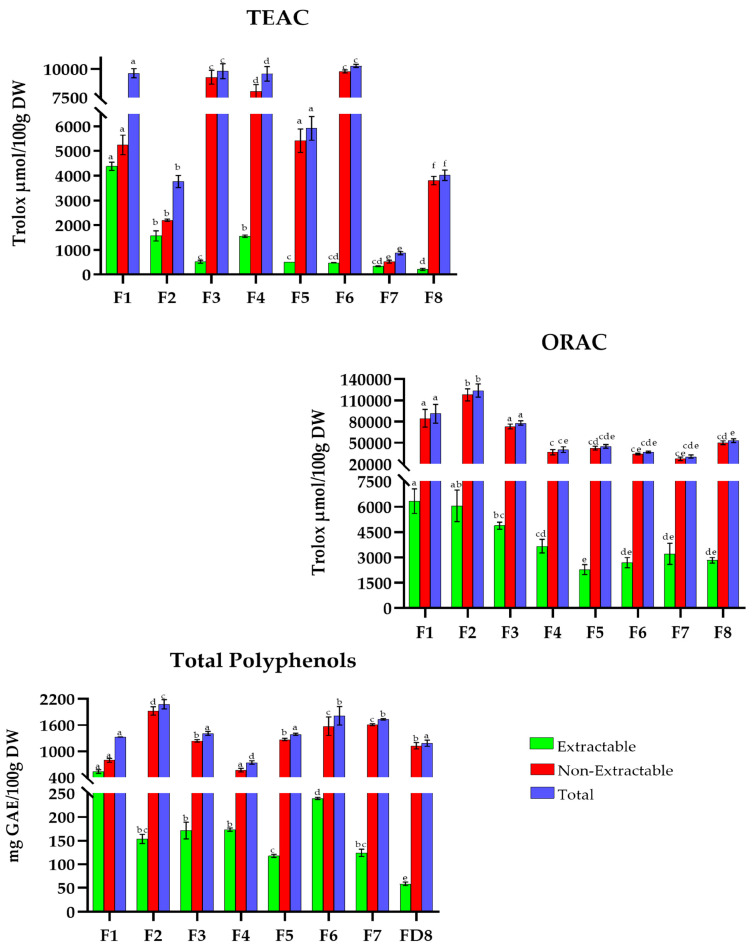
Total antioxidant capacity (TEAC and ORAC) and Total Polyphenols in traditional dishes in France (F1–F8). F1: Ratatouille; F2: Fish stew; F3: Cassoulet; F4: Basque pepper stew; F5: Meatballs with sauce; F6: French beef stew; F7: Tielle sétoise; F8: Basque cake. TEAC: Trolox Equivalent Absorbance Capacity; ORAC: Oxygen Radical Absorbance Capacity; GAEs: Gallic Acid Equivalents; DW: Dry Weight. Different letters (a–f) within the same color pattern indicate significant differences between samples (n = 3 replicates per sample (TEAC and TP), n = 4 replicates per sample (ORAC)), as determined by one-way ANOVA (*p* < 0.05).

**Figure 2:**
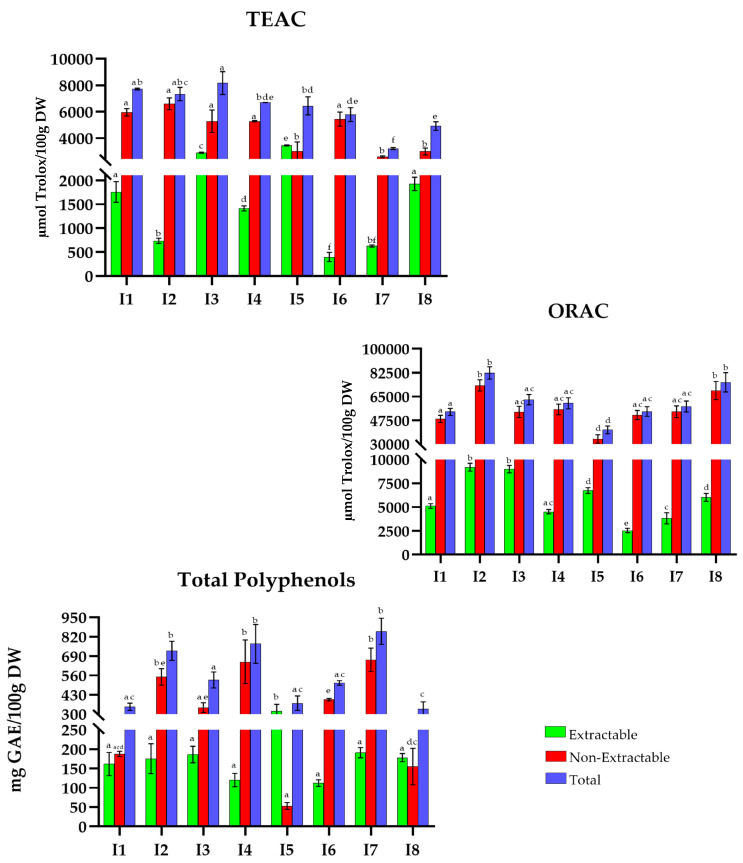
Total antioxidant capacity (TEAC and ORAC) and Total Polyphenols in traditional dishes in Italy (I1–I8). I1: Stewed dolphinfish; I2: Sardine meatballs; I3: Green olives pate; I4: Raw of sea bream and croaker fish with mango and green olives; I5: Sicilian caponata; I6: Bread and chickpea flour fritters; I7: Margherita pizza; I8: Tiramisu. TEAC: Trolox Equivalent Absorbance Capacity; ORAC: Oxygen Radical Absorbance Capacity; GAEs: Gallic Acid Equivalents; DW: Dry Weight. Different letters (a–f) within the same color pattern indicate significant differences between samples (n = 3 replicates per sample (TEAC and TP), n = 4 replicates per sample (ORAC)), as determined by one-way ANOVA (*p* < 0.05).

**Figure 3:**
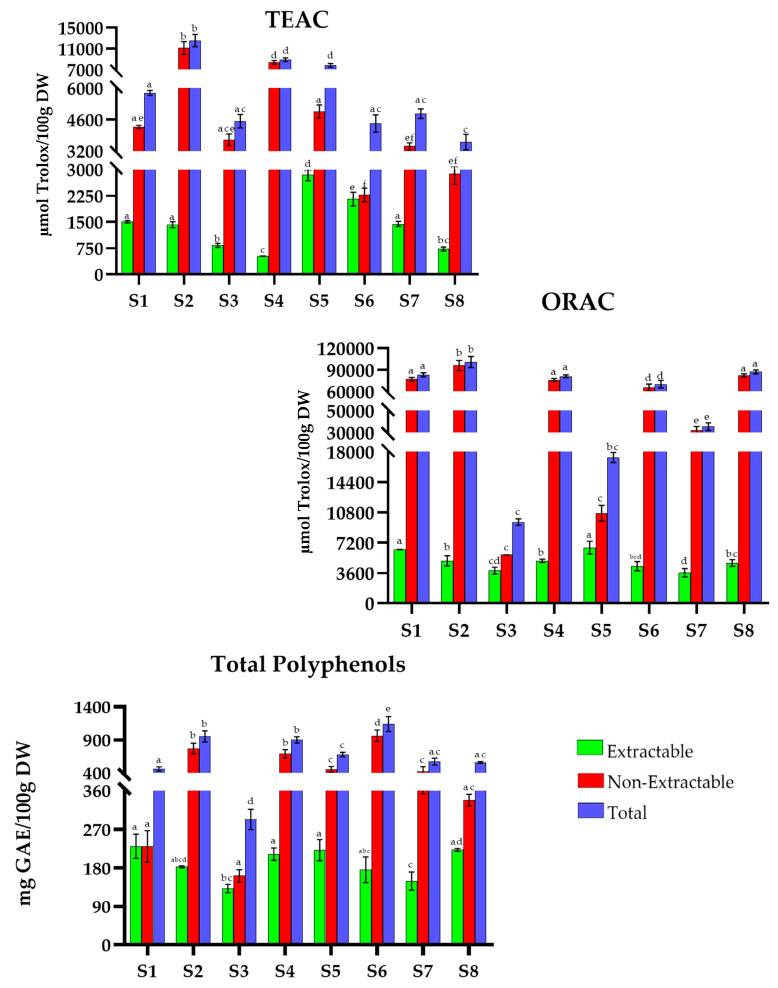
Total antioxidant capacity (TEAC and ORAC) and Total Polyphenols in traditional dishes in Spain (S1–S8). S1: Garlic rabbit; S2: Valencian hake; S3: Potato omelette; S4: Fish suquet; S5: Valencian titaina; S6: Zarangollo; S7: Valencian paella; S8: Torrija with horchata. TEAC: Trolox Equivalent Absorbance Capacity; ORAC: Oxygen Radical Absorbance Capacity; GAEs: Gallic Acid Equivalents; DW: Dry Weight. Different letters (a–f) within the same color pattern indicate significant differences between samples (n = 3 replicates per sample (TEAC and TP), n = 4 replicates per sample (ORAC)), as determined by one-way ANOVA (*p* < 0.05).

**Figure 4:**
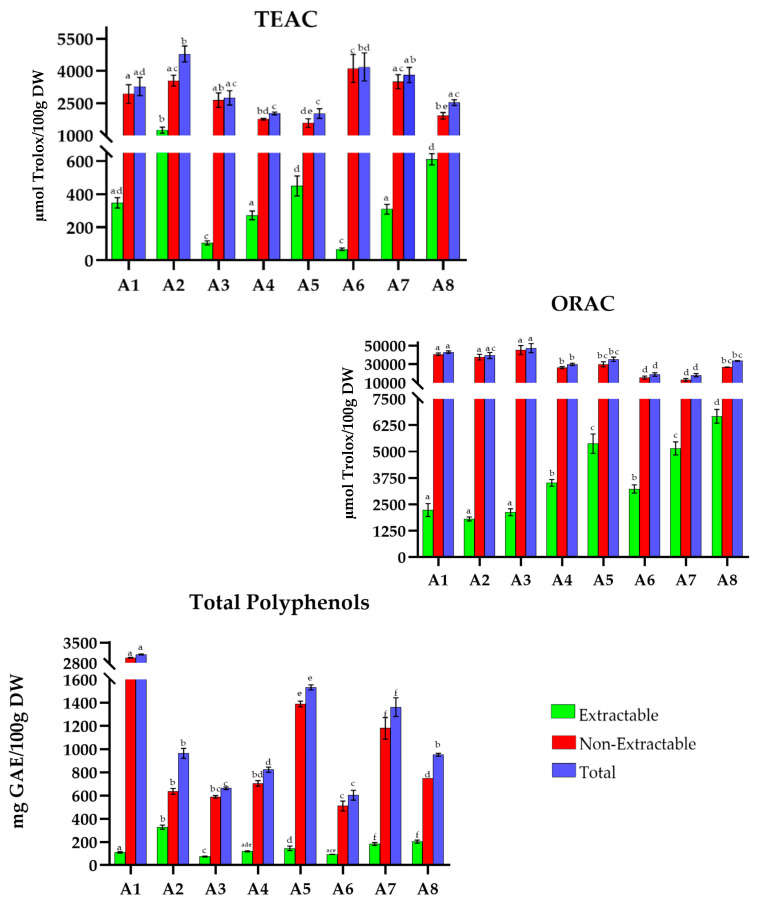
Total antioxidant capacity (TEAC and ORAC) and Total Polyphenols in traditional dishes in Algeria (A1–A8). A1: Rechta; A2: White chorba; A3: Mtewem; A4: Dobara; A5: Olive tagine; A6: Beans olive oil; A7: Sweet lamb; A8: Shakshuka. TEAC: Trolox Equivalent Absorbance Capacity; ORAC: Oxygen Radical Absorbance Capacity; GAEs: Gallic Acid Equivalents; DW: Dry Weight. Different letters (a–f) within the same color pattern indicate significant differences between samples (n = 3 replicates per sample (TEAC and TP), n = 4 replicates per sample (ORAC)), as determined by one-way ANOVA (*p* < 0.05).

**Figure 5:**
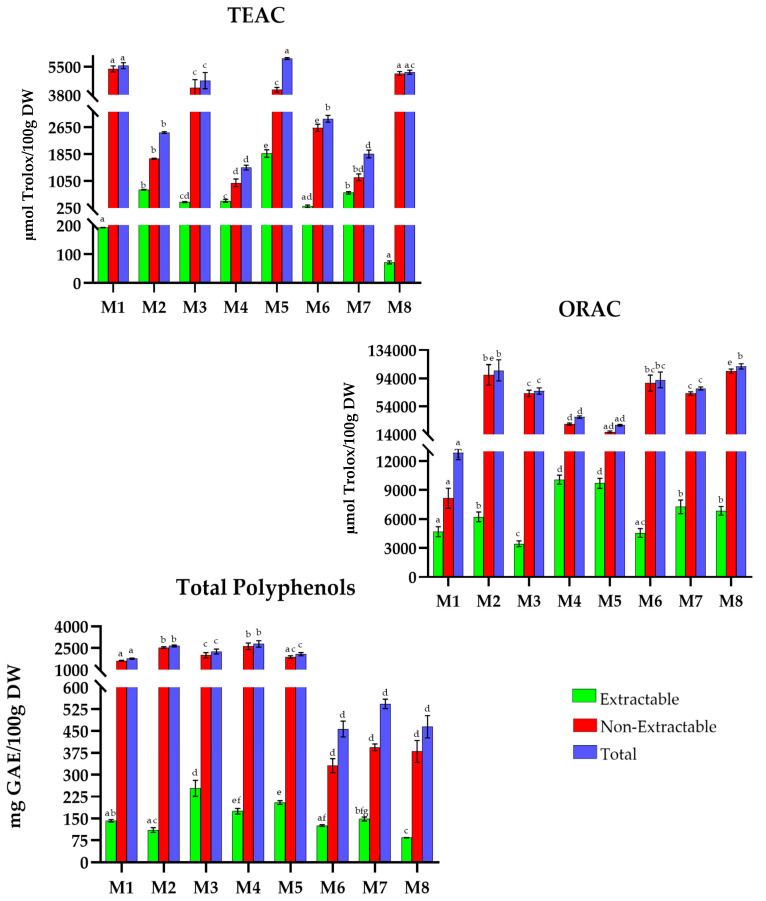
Total antioxidant capacity (TEAC and ORAC) and Total Polyphenols in traditional dishes in Morocco (M1–M8). M1: Bissara; M2: Harira; M3: Chicken vermicelli; M4: Chicken tagine with olive and preserved lemons; M5: Lamb/beef tagine with peas and artichokes; M6: Tagine with quinces and honey; M7: Beef tagine with apricots, prunes and almonds; M8: Minichicken bastille. TEAC: Trolox Equivalent Absorbance Capacity; ORAC: Oxygen Radical Absorbance Capacity; GAEs: Gallic Acid Equivalents; DW: Dry Weight. Different letters (a–g) within the same color pattern indicate significant differences between (n = 3 replicates per sample (TEAC and TP), n = 4 replicates per sample (ORAC)), as determined by one-way ANOVA (*p* < 0.05).

**Figure 6:**
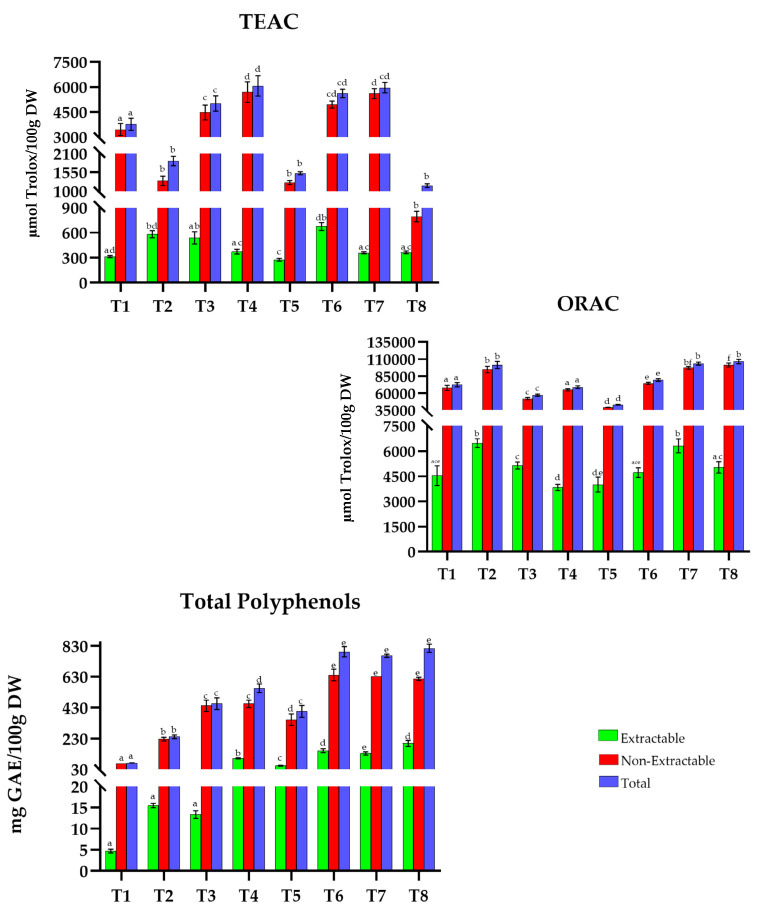
Total antioxidant capacity (TEAC and ORAC) and Total Polyphenols in traditional dishes in Tunisia (T1–T8). T1: Djerbian rice; T2: Tunisian lentil soup; T3: Ojja with merguez; T4: Kafteji; T5: Couscous with meat and vegetables; T6: Mloukhia; T7: Kamounia; T8: Assidat zgougou. TEAC: Trolox Equivalent Absorbance Capacity; ORAC: Oxygen Radical Absorbance Capacity; GAEs: Gallic Acid Equivalents; DW: Dry Weight. Different letters (a–f) within the same color pattern indicate significant differences between (n = 3 replicates per sample (TEAC and TP), n = 4 replicates per sample (ORAC)), as determined by one-way ANOVA (*p* < 0.05).

**Figure 7:**
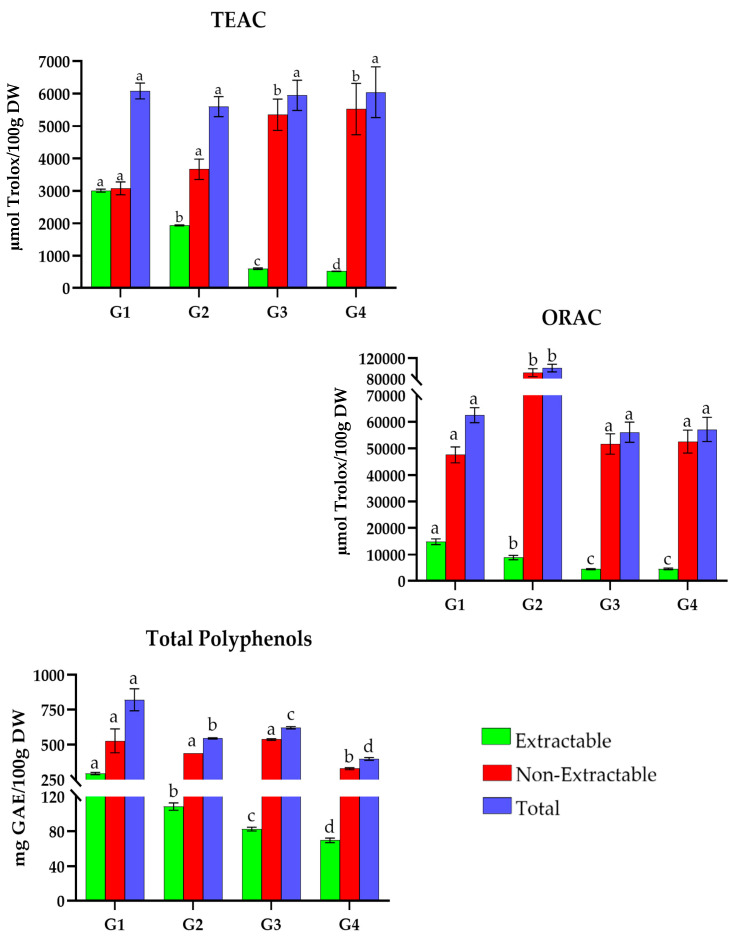
Total antioxidant capacity (TEAC and ORAC) and Total Polyphenols traditional dishes in Germany (G1–G4). G1: Gulasch; G2: Königsberger meatballs; G3: Pea soup; G4: Rice pudding with sugar and cinnamon. TEAC: Trolox Equivalent Absorbance Capacity; ORAC: Oxygen Radical Absorbance Capacity; GAEs: Gallic Acid Equivalents; DW: Dry Weight Different letters (a–d) within the same color pattern indicate significant differences between samples (n = 3 replicates per sample (TEAC and TP), n = 4 replicates per sample (ORAC)), as determined by one-way ANOVA (*p* < 0.05).

**Figure 8:**
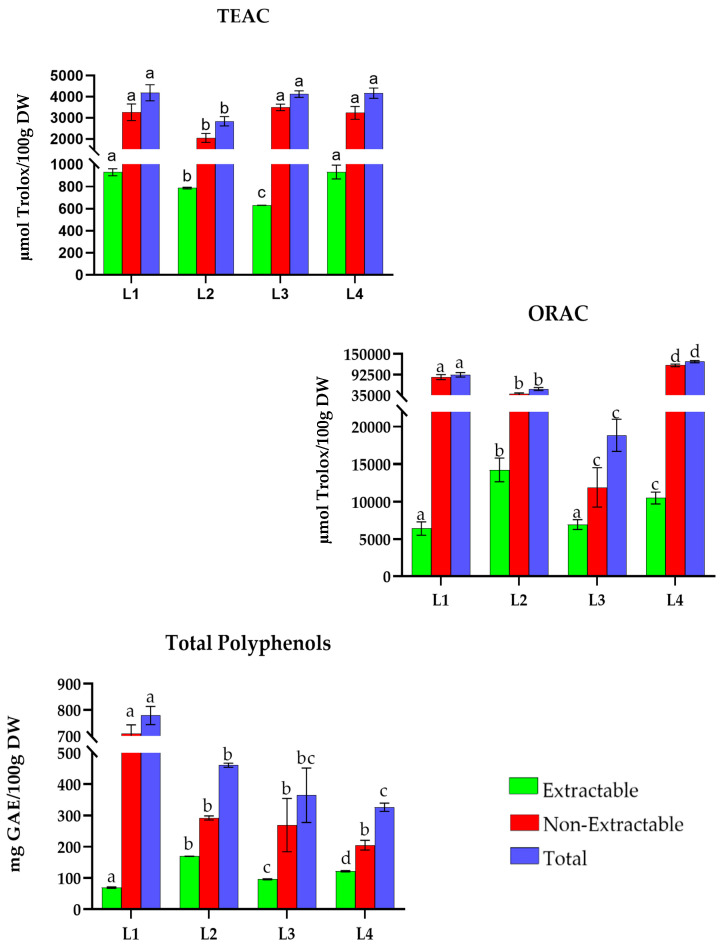
Total antioxidant capacity (TEAC and ORAC) and Total Polyphenols in traditional dishes in Luxembourg (L1–L4). L1: Judd with broad beans; L2: Green bean soup; L3: Plum tart; L4: Tarte tatin with vanilla ice cream. TEAC: Trolox Equivalent Absorbance Capacity; ORAC: Oxygen Radical Absorbance Capacity; GAEs: Gallic Acid Equivalents; DW: Dry Weight. Different letters (a–d) within the same color pattern indicate significant differences between samples (n = 3 replicates per sample (TEAC and TP), n = 4 replicates per sample (ORAC)), as determined by one-way ANOVA (*p* < 0.05).

The overall results obtained for TAC (TEAC and ORAC (µmol Trolox/100 g DW)) and TP (mg GAE/100 g DW) for traditional MD recipes are shown in Figure 1, Figure 2, Figure 3, Figure 4, Figure 5, Figure 6, Figure 7 and Figure 8. For the graphical representation of the results, each recipe has been coded (for example, in France: F1, F2, F3…) to improve readability. The correspondence between the codes and the recipes is detailed in the respective figure captions.

### 3.1. Total Antioxidant Capacity

#### 3.1.1. Mediterranean European Countries

The traditional recipes from France, Italy, and Spain show a wide variability for total (considering the sum of extractable and non-extractable antioxidants) antioxidant capacity. In the TEAC method, the values range from 860 to 12,600 µmol Trolox/100 g DW, while in the ORAC method, the values range from 9600 to 12,400 µmol Trolox/100 g DW, and TP values range between 290 and 2100 mg GAE/100 g DW.

The recipes in France (Figure 1), which range between 860 and 10,300 µmol Trolox/100 g DW in TEAC, show that the dishes with the highest TAC are *cassoulet* (F3) and *French beef stew* (F6) (9803.7 and 10,270.1 µmol Trolox/100 g DW, respectively), in contrast *tielle sétoise* (F7) provides the lowest (868.1 µmol Trolox/100 g DW).

In the ORAC method, the ranges fluctuate between 30,000 and 124,000 µmol Trolox/100 g DW, *fish stew *(F2) with 123,918.9 µmol Trolox/100 g DW, while the lowest TACs are for *meatballs with sauce *(F5), *French beef stew *(F6), and *tielle sétoise *(F7) (45,103.1, 37,111.1, and 30,744.3 µmol Trolox/100 g DW, respectively).

In TP, the values fluctuate between 740 and 2100 mg GAE/100 g DW, with *fish stew *(F2) being the recipe with the highest total phenolic content (2078.7 mg GAE/100 g DW) and *basque pepper stew* (F4) the lowest (742.5 mg GAE/100 g DW).

In Italy (Figure 2), the range is between 3200 and 8200 µmol Trolox/100 g DW in the TEAC method. The dishes with the highest TAC are *stewed dolphinfish *(I1), *sardine meatballs *(I2), and *green olives pate *(I3) with 7702.6, 7325.1, and 8158.4 µmol Trolox/100 g DW, respectively. On the contrary, *margherita pizza *(I7) (3208.3 µmol Trolox/100 g DW) shows the lowest total TEAC values.

Concerning the ORAC method, the highest total TAC are for the recipes *sardine meatballs *(I2) and *tiramisú* (I8) (82,365.7 and 75,438.3 µmol Trolox/100 g DW), except *Sicilian caponata *(I5) with 40,477.6 µmol Trolox/100 g DW, which displays the lowest values.

Furthermore, Italian recipes display a more moderate phenolic content, ranging from 350 to 860 mg GAE/100 g DW. In this group, the recipes with the highest total polyphenol content are *margherita pizza* (I7); *raw sea bream with croaker fish, mango, and green olives *(I4) (853.6 mg GAE/100 g DW); and *sardine meatballs *(I2) (856.8, 853.6, and 747.7 mg GAE/100 g DW, respectively). In contrast, preparations such as *stewed dolphinfish *(I1) or *tiramisú* (ID8) have a lower value (366.7 and 357.40 mg GAE/100 g DW, respectively).

In Spain (Figure 3), the values range from 3600 to 12,600 µmol Trolox/100 g DW in TEAC, with *Valencian hake* (S2) being the recipe with the highest antioxidant activity recipe (12,552.7 µmol Trolox/100 g DW). *Potato omelette *(S3), *zarangollo *(S6), *Valencian paella *(S7), and *torrija with horchata *(S8) represent the recipes with the lowest antioxidant capacity (4531.6, 4431.1, 4859.0, and 3606.4 µmol Trolox/100 g DW, respectively).

Considering the ORAC method, the values range from 9600 to 102,000 µmol Trolox/100 g DW. *Valencian hake *(S2) (101,066.3 µmol Trolox/100 g DW) is the recipe with the highest total ORAC, while *potato omelette *(S3) and *valencian titaina *(S5) show the lowest values (9645.6 and 17,118.9 µmol Trolox/100 g DW, respectively).

Regarding TP, the recipes show intermediate values within Mediterranean European countries, ranging from 290 to 1200 mg GAE/100 g DW. *Zarangollo *(S6) stands out (1121.0 mg GAE/100 g DW). However, *Potato omelette* (S3) has the lowest phenolic content (293.1 mg GAE/100 g DW).

#### 3.1.2. Mediterranean African Countries

The traditional recipes of Argelia, Morocco, and Tunisia exhibit TAC values ranging from 1100 to 6100 µmol Trolox/100 g DW in TEAC and from 12,000 to 111,200 µmol Trolox/100 g DW in ORAC, while for TP values ranging between 70 and 3100 mg GAE/100 g DW.

Algerian recipes (Figure 4) show TAC values ranging from 2000 to 4800 µmol Trolox/100 g DW in TEAC. Dishes such as *white chorbak* (A2), *beans with olive oil *(A6), and *sweet lamb *(A7) show the highest values with 4789.6, 4178.1, and 3807.9 µmol Trolox/100 g DW, respectively; while *dobara *(A4), *olive tajine *(A5), and *shakshuka* (A8) have the lowest values (1748.5, 1567.8 and 1914.7 µmol Trolox/100 g DW).

About the ORAC method, values range between 18,200 and 47,300 µmol Trolox/100 g DW. Dishes such as *rechta* (A1), *white chorba *(A2), and *mtewem *(A3) show the highest values with 42,783.3, 39,144.5, and 47,200.7 µmol Trolox/100 g DW, respectively. *Beans with olive oil* (A6) and *sweet lamb *(A7) show the lowest total ORAC values (18,844.1 and 18,224.0 µmol Trolox/100 g DW).

Considering TP, recipes show a range from 600 to 3100 mg GAE/100 g DW, with *rechta* (A1) leading at 3082.2 mg GAE/100 g DW, while *mtewem* (A3) and *beans with olive oil* (A6) have the lowest values at 662.5 and 602.6 mg GAE/100 g DW, respectively.

Moroccan dishes (Figure 5), for the TEAC method, present values ranging from 1400 to 6000 µmol Trolox/100 g DW. Dishes such as *bissara *(M1), *lamb/beef tagine with peas and artichokes* (M5) and *minichicken bastilla* (M8) have the highest total TAC (5553.3, 5980.0, and 5164.3 µmol Trolox/100 g DW). On the other hand, *chicken tagine with olives and lemons *(M4) and *beef tagine with apricots, prunes, and almonds *(M7) with 1444.4 and 1850 µmol Trolox/100 g DW represent the recipes with the lowest capacity.

In the ORAC case, values range between 12,800 and 112,000 µmol Trolox/100 g DW. Recipes such as *harira *(M2), *tagine with quinces and honey *(M6) and *minichicken bastilla *(M8) have the highest capacity with total values of 105,272.8, 91,833.9, and 111,101.7 µmol Trolox/100 g DW, respectively, with *bissara *(M1) showing the lowest one (12,839.6 µmol Trolox/100 g DW).

Regarding TP, the values range from 450 to 2800 mg GAE/100 g DW, with *harira* (M2) and *chicken tagine with olives and preserved lemons* (M4) (2614.8 and 2786.3 mg GAE/100 g DW, respectively) standing out. In contrast, *tagine quinces and honey* (M6), *beef tagine with apricots, prunes, and almonds* (M7), and *mini chicken bastilla* (M8) have the lowest phenolic content, with values of 456.9, 542.7, and 464.5 mg GAE/100 g DW, respectively.

Tunisian recipes (Figure 6) show TAC values that range between 1100 and 6100 µmol Trolox/100 g DW in the TEAC method. Dishes such as *kafteji *(T4), *mloukhia *(T6), and *kamounia *(T7) show the highest values (6067.6, 5623.6, and 5963 µmol Trolox/100 g DW, respectively). In contrast, *tunisian lentill soup* (T2), *couscous with meat and vegetables* (T5), and *assidat zgougou *(T8) represent the recipes with the lowest content (1884.7, 1526.6, and 1160.3 µmol Trolox/100 g DW).

In the case of the ORAC method, recipes exhibit values ranging from 43,000 to 106,500 µmol Trolox/100 g DW. Recipes such as *tunisian lentill soup *(T2), *kamounia *(T7), and *assidat zgougou* (T8) have the highest TAC (101,419.3, 103,592.0, and 106,494.9 µmol Trolox/100 g DW, respectively), while *couscous with meat and vegetables* (T5) has the lowest capacity (43,118.9 µmol Trolox/100 g DW).

Finally, TP values range from 70 to 815 mg GAE/100 g DW, with *mloukhia* (T6), *kamounia* (T7), and *assidat zgougou* (T8) standing out at 791.7, 765.5, and 814.2 mg GAE/100 g DW, respectively. In contrast, *djerbian rice* (T1) has the lowest phenolic content in the region, with 71.5 mg GAE/100 g DW.

#### 3.1.3. Non-Mediterranean European Countries

The traditional recipes of Germany and Luxembourg exhibit TAC values ranging from 4100 to 6100 µmol Trolox/100 g DW in TEAC and from 18,800 to 130,000 µmol Trolox/100 g DW in ORAC, whereas for the TP values range between 320 to 830 mg GAE/100 g DW.

German recipes (Figure 7) display TAC values ranging from 5500 and 6100 µmol Trolox/100 g DW with the TEAC method. No significant differences are observed for total values between the recipes.

Considering the ORAC method, total TAC values vary between 56,000 to 101,000 µmol Trolox/100 g DW. *Königsberger meatballs *(G2) possess the highest value with 100,773.2 µmol Trolox/100 g DW. The lowest values are for the recipes *gulasch *(G1), *pea soup *(G3), and *rice pudding with sugar and cinnamon *(G4) with 62,492.8, 56,084.9, and 57,132.2 µmol Trolox/100 g DW, respectively.

For TP, recipes show a range between 390 and 830 mg GAE/100 g DW, with *gulasch* (G1) as a higher phenolic-containing dish (821.6 mg GAE/100 g DW), while *rice pudding with sugar and cinnamon *(G4) shows the lowest values (398.6 mg GAE/100 g DW).

Luxembourg recipes (Figure 8) reveal TAC values ranging from 2800 to 4200 µmol Trolox/100 g DW with the TEAC method. *Judd with broad beans *(L1), *plum tart *(L3), and *tarte tatin with vanilla ice cream *(L4) show the highest total TAC values (4190.6, 4123.0, and 4166.5 µmol Trolox/100 g DW). In contrast, *green bean soup *(L2) is the dish with the lowest TAC, with 2836.7 µmol Trolox/100 g DW.

Considering the ORAC method, values of recipes range between 18,000 and 130,000 µmol Trolox/100 g DW. *Tarte tatin with vanilla ice cream *(L4) presents the highest TAC (128,585.7 µmol Trolox/100 g DW). On the other hand, *plum tart *(L3) is the recipe with the lowest value (18,847.5 µmol Trolox/100 g DW).

Finally, TP values range from 320 to 780 mg GAE/100 g DW, with *judd with broad beans* (L1) standing out at 778.4 mg GAE/100 g DW. In contrast, *plum tart *(L3) and *tarte tatin with vanilla ice cream *(L4) have the lowest phenolic content in the region, with 413.2 and 326.1, respectively.

### 3.2. Extractable and Non-Extractable Fractions

#### 3.2.1. Mediterranean European Countries

The percentage contribution of extractable and non-extractable fractions was calculated for each recipe for Mediterranean European countries relative to the total value of each individual assay (Table 1). In France (Figure 1), the values of extractable antioxidants, measured through TEAC, ORAC, and TP, ranged from 210.7 to 4383.3 µmol Trolox/100 g DW, 2275.7 to 6324 µmol Trolox/100 g DW, and 58.3 to 534.7 mg GAE/100 g DW, respectively. *Ratatouille *(F1) stood out in TEAC and TP methodologies, with 46% and 40%, respectively, while *Tielle sétoise* (F7) distinguished itself in the ORAC method with 10%. On the other hand, the non-extractable fraction exhibited values between 525.3 and 9787.7 µmol Trolox/100 g DW (TEAC), 27,535.9 and 117,856.3 µmol Trolox/100 g DW (ORAC), and 569.2 and 793.9 mg GAE/100 DW (TP), where *basque cake *(F8) had the highest values (95% in all three methods).

In Italy (Figure 2), the values of extractable antioxidant capacity ranged from 342.9 to 3441.8 µmol Trolox/100 g DW (TEAC), 2537.0 to 9197.7 µmol Trolox/100 g DW (ORAC), and 112.3 to 320.3 mg GAE/100 g DW (TP). *Sicilian caponata* (I5) showed the highest values in TEAC (53%), ORAC (17%), and TP (86%). The non-extractable fraction varied between 2580.4 and 6590.5 µmol Trolox/100 g DW (TEAC), 33,778.7 to 73,168 µmol Trolox/100 g DW (ORAC), and 112.3 to 320.3 mg GAE/100 g DW (TP). In this fraction, *bread and chickpea flour fritters* (I6) reached 94% (TEAC) and 95% (ORAC), while *raw sea bream and croaker fish with mango and green olives *(I4) stood out with 86% in TP.

In Spain (Figure 3), the values of extractable antioxidants ranged from 520.1 to 2848.4 µmol Trolox/100 g DW (TEAC), 3628.7 to 6403.7 µmol Trolox/100 g DW (ORAC), and 131.6 to 230.2 mg GAE/100 g DW (TP). *Zarangollo *(S6) (49%), *potato omelette *(S3) (40%), and *garlic rabbit* (S1) (50%) stood out in TEAC, ORAC, and TP, respectively. The non-extractable fraction ranged from 2275.1 to 11,130 µmol Trolox/100 g DW (TEAC), 5754.1 to 96,005.5 µmol Trolox/100 g DW (ORAC), and 161.4 to 962.5 mg GAE/100 g DW (TP). In this fraction, *fish suquet* (S4) showed the highest value (94%) in TEAC, *Valencian hake *(S2) and *Torrija with horchata* (S8) (95%) in ORAC, and *zarangollo* (S6) in TP (86%).

**Table 1:** Contribution (%) of extractable and non-extractable antioxidant compounds of Mediterranean European countries’ dishes.

Mediterranean European Countries	Dishes	TEAC		ORAC		Total Polyphenols	
		Extractable	Non- Extractable	Extractable	Non- Extractable	Extractable	Non- Extractable
France	F1	46	54	7	93	40	60
	F2	42	48	5	95	12	88
	F3	5	95	6	94	12	88
	F4	16	84	9	91	23	77
	F5	8	92	5	95	8	92
	F6	5	95	7	93	13	87
	F7	39	61	10	90	7	93
	F8	5	95	5	95	5	95
Italy	I1	23	77	10	90	49	51
	I2	10	90	11	89	26	74
	I3	35	65	14	86	35	65
	I4	19	81	8	92	14	86
	I5	53	47	17	83	86	14
	I6	6	94	5	95	22	78
	I7	20	80	7	93	22	78
	I8	39	61	8	92	50	50
Spain	S1	26	74	8	92	50	50
	S2	11	89	5	95	19	81
	S3	18	82	40	60	45	55
	S4	6	94	6	94	24	76
	S5	36	64	37	63	33	67
	S6	49	51	6	94	14	86
	S7	30	70	10	90	26	74
	S8	20	80	5	95	40	60

F1: Ratatouille; F2: *Fish stew*; F3: *Cassoulet*; F4: *Basque pepper stew*; F5: *Meatballs with sauce*; F6: *French beef stew*; F7: *Tielle sétoise*; F8: *Basque cake*; I1: *Stewed dolphinfish*; I2: *Sardine meatballs*; I3: *Green olives pate*; I4: *Raw of sea bream and croaker fish with mango and green olives*; I5: *Sicilian caponata*; I6: *Bread and chickpea flour fritters*; I7: *Margherita pizza*; I8: *Tiramisu*; S1: *Garlic rabbit*; S2: *Valencian hake*; S3: *Potato omelette*; S4: *Fish suquet*; S5: *Valencian titaina*; S6: *Zarangollo*; S7: *Valencian paella*; S8: *Torrija with horchata*. TEAC: Trolox Equivalent Antioxidant Capacity; ORAC: Oxygen Radical Absorbance Capacity.

#### 3.2.2. Mediterranean African Countries

The percentage contribution of extractable and non-extractable fractions was calculated for each recipe from Mediterranean African countries relative to the total value of each individual assay Table 2. In Algeria (Figure 4), extractable antioxidants ranged from 66.7 to 1243.6 µmol Trolox/100 g DW in TEAC, 1812.4 to 6673.1 µmol Trolox/100 g DW in ORAC, and 73.7 to 327.3 mg GAE/100 g DW in TP. *White chorba *(A2) (26% in TEAC and 34% in TP) and *sweet lamb* (A7) (28%) were the most notable. The non-extractable fraction showed values between 1567.8 and 4111.5 µmol Trolox/100 g DW (TEAC), 13,071.1 to 45,072.5 µmol Trolox/100 g DW (ORAC), and 509.9 to 2972.2 mg GAE/100 g DW (TP), with *couscous with beans and olive oil* (A6) (98%, TEAC), *rechta* (A1), *white chorba *(A2), and *mtewem* (A3) (95%, ORAC), and *rechta* (A1) (96%, TP) leading in each method.

In Morocco (Figure 5), extractable antioxidant values ranged from 71.8 to 1866.5 µmol Trolox/100 g DW in TEAC, 3432.5 to 15,535.2 µmol Trolox/100 g DW in ORAC, and 84.1 to 253.6 mg GAE/100 g DW in TP. *Beef tagine with apricots, prunes, and almonds *(M7) (37%, TEAC), *lamb/beef tagine with peas and artichokes* (M5) (42%, ORAC), and *tagine with quinces and honey *(M6) (28%, TP) were the most outstanding. The non-extractable fraction varied between 989.6 and 5361.0 µmol Trolox/100 g DW, 8155.1 to 99,052.5 µmol Trolox/100 g DW, and 330.9 to 2611.1 mg GAE/100 g DW for TEAC, ORAC, and TP, respectively, with *mini chicken bastilla* (M8) (99%, TEAC), *chicken vermicelli* (M3) *and tagine quinces and honey* (M6) (95%, ORAC), and *harira *(M2) (96%, TP) standing out.

In Tunisia (Figure 6), the values of extractable antioxidants ranged from 276.0 to 675.5 µmol Trolox/100 g DW in TEAC, 3834.4 to 6472.6 µmol Trolox/100 g DW in ORAC, and 4.7 to 199.0 mg GAE/100 g DW in TP. *Assidat zgougou* (T8) (32% in TEAC and 24% in TP), *ojja with merguez *(T3), and *couscous with meat and vegetables* (T5) (9% in ORAC) were the most notable. In the non-extractable fraction, the values ranged from 4948.1 to 5695.4 µmol Trolox/100 g DW in TEAC, 39,107.8 to 101,466.4 µmol Trolox/100 g DW in ORAC, and 66.8 to 640.4 mg GAE/100 g DW in TP. *Kafteji *(T4) and *kamounia* (T7) (94%, TEAC), *assidat zgougou* (T8) (95%, ORAC), and *ojja with merguez* (T3) (97%, TP) were the most outstanding.

**Table 2:** Contribution (%) of extractable and non-extractable antioxidant compounds of Mediterranean African countries’ dishes.

Mediterranean African Countries	Dishes	TEAC		ORAC		Total Polyphenols	
		Extractable	Non Extractable	Extractable	Non Extractable	Extractable	Non Extractable
Algeria	A1	11	89	5	95	4	96
	A2	26	74	5	95	34	66
	A3	4	96	5	95	11	89
	A4	13	87	12	88	14	86
	A5	22	78	15	85	9	91
	A6	2	98	17	83	15	85
	A7	8	92	28	72	13	87
	A8	24	76	20	80	21	79
Morocco	M1	3	97	36	64	8	92
	M2	35	65	6	94	4	96
	M3	9	91	5	95	11	89
	M4	31	69	35	65	6	94
	M5	31	69	42	58	10	90
	M6	9	91	5	95	28	72
	M7	37	63	9	91	27	73
	M8	1	99	6	94	18	82
Tunisia	T1	8	92	6	94	7	93
	T2	31	69	6	94	6	94
	T3	11	89	9	91	3	97
	T4	6	94	6	94	18	82
	T5	18	82	9	91	13	87
	T6	12	88	6	94	19	81
	T7	6	94	6	94	17	83
	T8	32	68	5	95	24	76

A1: *Rechta*; A2: *White chorba*; A3: *Mtewem*; A4: *Dobara*; A5: *Olive tagine*; A6: *Beans olive oil*; A7: *Sweet lamb*; A8: *Shakshuka*; M1: *Bissara*; M2: *Harira*; M3: *Chicken vermicelli*; M4: *Chicken tagine with olive and preserved lemons*; M5: *Lamb/beef tagine with peas and artichokes*; M6: *Tagine with quinces and honey*; M7: *Beef tagine with apricots, prunes and almonds*; M8: *Minichicken bastille*; T1: *Djerbian rice*; T2: *Tunisian lentil soup*; T3: *Ojja with merguez*; T4: *Kafteji*; T5: *Couscous with meat and vegetables*; T6: *Mloukhia*; T7: *Kamounia*; T8: *Assidat zgougou*; TEAC: Trolox Equivalent Antioxidant Capacity; ORAC: Oxygen Radical Absorbance Capacity.

#### 3.2.3. Non-Mediterranean European Countries

The percentage contribution of extractable and non-extractable fractions was calculated for each recipe from non-Mediterranean European countries relative to the total value of each individual assay (Table 3). In Germany (Figure 7), extractable antioxidants ranged from 515.9 to 3007.4 µmol Trolox/100 g DW in TEAC, 4433.0 to 14,869.5 µmol Trolox/100 g DW in ORAC, and 69.9 to 293.9 mg GAE/100 g DW in TP. *Gulasch* (G1) stood out with 49%, 24%, and 36%, respectively. In the non-extractable fraction, values ranged from 3664.4 to 5524.9 µmol Trolox/100 g DW (TEAC), 47,623.3 to 91,904.8 µmol Trolox/100 g DW (ORAC), and 328.7 to 538.4 mg GAE/100 g DW (TP). In this fraction, *rice pudding with sugar and cinnamon* (G4) achieved 91% and 92% in TEAC and ORAC, while *pea soup* (G3) scored 87% in TP.

In Luxembourg (Figure 8), extractable antioxidant values ranged from 631.2 to 929.1 µmol Trolox/100 g DW in TEAC, 6404.3 to 14,238.4 µmol Trolox/100 g DW in ORAC, and 68.8 to 169.0 mg GAE/100 g DW in TP. *Green bean soup* (L2) (28%, TEAC), *plum tart* (L3) (37%, ORAC), and *green bean soup* (L2) and *tarte tatin with vanilla ice cream* (L4) (37%, TP) were the most notable. In the non-extractable fraction, values ranged from 2050.4 to 3235.6 µmol Trolox/100 g DW in TEAC, 11,916.6 to 118,090.3 µmol Trolox/100 g DW in ORAC, and 204.5 to 709.7 mg GAE/100 g DW in TP. *Plum tart *(L3) stood out with 86% in TEAC, while in ORAC and TP, *judd with broad beans* (L1) reached 93% and 91%, respectively.

**Table 3:** Contribution (%) of extractable and non-extractable antioxidant compounds of non-Mediterranean European countries’ dishes.

No Mediterranean European Countries	Dishes	TEAC		ORAC		Total Polyphenols	
		Extractable	Non Extractable	Extractable	Non Extractable	Extractable	Non Extractable
Germany	G1	49	51	24	76	36	64
	G2	34	66	9	91	20	80
	G3	10	90	14	86	13	87
	G4	9	91	8	92	18	82
Luxembourg	L1	22	78	7	93	9	91
	L2	28	72	24	76	37	63
	L3	14	86	37	63	23	77
	L4	22	78	8	92	37	63

G1: *Gulasch*; G2: *Königsberger meatballs*; G3: *Pea soup*; G4: *Rice pudding with sugar and cinnamon*; L1: *Judd with broad beans*; L2: *Green bean soup*; L3: *Plum tart*; L4: *Tarte tatin with vanilla ice cream*; TEAC: Trolox Equivalent Antioxidant Capacity; ORAC: Oxygen Radical Absorbance Capacity.

### 3.3. Phenolic Compounds Profiling

#### 3.3.1. Qualitative Characterization of the Phenolic Composition

A representative base peak chromatogram (BPC) of the composite extract is shown in Figure S1 (Supplementary Materials), with detected peaks numbered according to their elution order. The analytical characteristics for each peak, including retention time, molecular formula, experimental *m*/*z*, mass error, and mSigma values, are summarized in Table S1 (Supplementary Materials).

The chromatographic analysis revealed a broad diversity of phenolic compounds, including several chemical families such as simple phenols, phenolic acids, flavonoids, flavan-3-ols, lignans, anthocyanins, and secoiridoid derivatives. This diversity reflects the phytochemical complexity of the plant-derived ingredients used in the analyzed recipes and aligns with the wide variety of polyphenols typically found in foods associated with the Mediterranean diet. Polyphenols are widely distributed in fruits, vegetables, legumes, cereals, olive oil, and wine, which are the major components of Mediterranean dietary patterns [48,49].

A total of 36 compounds were detected and annotated across the chromatographic profiles of the analyzed samples, representing a broad diversity of phenolic families. These included mainly phenolic acids (10), flavonols (4), anthocyanins (4), secoiridoids (3), simple phenols (2), flavan-3-ols (2), flavones (2), and flavonol glycosides (2). In addition, several minor subclasses were represented by a single compound each: a flavanone, a flavone glycoside, a phenylpropanoid glycoside, a condensed tannin, a stilbene, and a lignan. The occurrence of these compounds varied among samples, reflecting differences in ingredient composition and culinary practices among the studied recipes. Besides these phenolic constituents, citric acid was also detected in the chromatographic profiles; however, this compound belongs to the class of organic acids rather than phenolic compounds, although it was detected under the same analytical conditions.

Beyond this overall diversity, the qualitative distribution of phenolic compounds suggested distinct compositional tendencies based on the country of origin of the recipes and their culinary traditions. Recipes originating from Mediterranean European countries showed a notable presence of olive-related phenolics, including hydroxytyrosol, tyrosol, and secoiridoid derivatives such as oleuropein, oleacein, and oleocanthal. These compounds are characteristic constituents of olive matrices and reflect the central role of olive oil as the primary culinary fat in many Mediterranean European countries. Olive-derived phenolics have been widely reported as important contributors to the antioxidant and anti-inflammatory properties associated with Mediterranean dietary patterns [50]. In addition, samples from Mediterranean European countries frequently contained flavonoids such as quercetin, luteolin, apigenin, and kaempferol, which are commonly associated with vegetables, fruits, and aromatic herbs widely used in Southern European cuisines. Flavonoids are one of the largest groups of dietary polyphenols and are recognized as key bioactive compounds present in Mediterranean plant foods [49,51]. In contrast, recipes from Mediterranean African countries showed a higher occurrence of phenolic acids, including caffeic acid, ferulic acid, p-coumaric acid, protocatechuic acid, and syringic acid. These compounds are widely distributed in vegetables, legumes, and spices, which play an important role in North African culinary traditions. Their presence may therefore reflect the significant contribution of pulses, vegetables, and aromatic spices to the phenolic composition of these recipes.

Across recipes from different countries, several compounds were shared components of the phenolic fraction, particularly flavonoids and flavan-3-ols such as catechin and epicatechin. These compounds are widely present in plant-based foods, including fruits, legumes, and cereals commonly consumed throughout Mediterranean countries, and are known to contribute significantly to the antioxidant potential of these foods [52]. The detection of lignans such as pinoresinol further supports the contribution of olive-derived ingredients and other plant matrices to the phenolic profile. Additionally, the occurrence of anthocyanins, including cyanidin and pelargonidin derivatives, in some recipes indicates the presence of pigmented plant ingredients such as fruits or colored vegetables, which are recognized sources of these compounds.

#### 3.3.2. Mediterranean European Countries

The European Mediterranean countries exhibited the highest phenolic contents among all the regions studied, largely dominated by phenolic acids and flavonoids (See Tables S2–S4 in Supplementary Materials). Within this region, phenolic acids were the most abundant class, with mean values fluctuating between 14.63 and 713.12 mg/kg DW in the French samples (*French beef stew* (F6) and *ratatouille* (F1), respectively), 4.85 and 500.43 mg/kg DW in the Italian samples (*Green olives pate* (I3) and *Margherita pizza* (I7), respectively), and, finally, 35.96 and 395.298 mg/kg DW for the Spanish counterparts (*Torrija with horchata* (S8) and *Potato omelette* (S3), respectively) (Table 4). Secoiridoids, characteristic of olive-based products, were also present in considerable levels, particularly in the Italian samples with a highest value of 465.87 mg/kg DW in the recipe *Green olives pate* (I3). In the Spanish studied samples, all the samples exhibited considerable contents of secoiridoids ranging from 7.36 to 50.33 mg/kg DW, while these compounds were not detected in 3 French recipes and fluctuated between 23.50 and 115.17 mg/kg DW in the other 5 recipes. Likewise, simple phenols were more abundant in the Italian recipes, with a highest value of around 307.03 mg/kg DW (*Green olives pate* (I3)) while these compounds were found in the French and Spanish samples in moderate contents.

Flavonols and their glycosides were present at moderate levels (with overall average values of 6.93 and 14.66 mg/kg DW, respectively), while flavanones (up to 31.77 mg/kg DW) and anthocyanins (up to 123.77 mg/kg DW) showed substantial variation depending on the country. Lignans were detected at low but consistent levels (less than 10 mg/kg DW), whereas stilbenes and phenylpropanoid glycosides were almost negligible. Therefore, the European Mediterranean region seems to display a rich and diverse phenolic spectrum in their recipes dominated by phenolic acids, flavonoids, and secoiridoids.

**Table 4:** Average contents (in mg/kg of DW) of phenolic compounds, expressed as the sum of each phenolic family, in recipes belonging to the European Mediterranean countries under study.

	France								Italy								Spain							
Phenolic Family	F1	F2	F3	F4	F5	F6	F7	F8	I1	I2	I3	I4	I5	I6	I7	I8	S1	S2	S3	S4	S5	S6	S7	S8
Phenolic acids	713.12 ± 1.21 a	379.89 ± 0.88 b	54.12 ± 0.34 c	26.01 ± 0.19 d	217.63 ± 0.67 e	14.63 ± 0.11 f	396.57 ± 0.92 b	129.27 ± 0.55 g	164.06 ± 0.73 a	148.95 ± 0.66 b	4.85 ± 0.06 c	74.20 ± 0.41 d	278.35 ± 0.84 e	375.78 ± 0.91 f	500.43 ± 1.03 g	283.52 ± 0.77 e	247.18 ± 0.71 a	259.10 ± 0.74 a	395.98 ± 0.97 b	93.82 ± 0.39 c	101.91 ± 0.42 d	269.58 ± 0.80 e	90.92 ± 0.37 f	35.96 ± 0.23 g
Simple phenols	5.34 ± 0.07 a	1.09 ± 0.03 b	ND	2.99 ± 0.05 c	10.02 ± 0.09 d	ND	1.97 ± 0.04 e	ND	37.18 ± 0.19 a	7.66 ± 0.08 b	307.03 ± 0.82 c	21.26 ± 0.18 d	43.85 ± 0.22 a	ND	0.28 ± 0.01 e	ND	21.87 ± 0.17 a	34.42 ± 0.21 b	2.33 ± 0.04 c	1.02 ± 0.02 d	1.39 ± 0.03 e	1.30 ± 0.03 e	1.02 ± 0.02 d	6.97 ± 0.07 f
Flavan-3-ols	ND	23.05 ± 0.21 a	18.01 ± 0.16 b	ND	35.41 ± 0.27 c	ND	ND	8.44 ± 0.09 d	4.38 ± 0.06 a	ND	ND	ND	ND	7.75 ± 0.08 b	ND	41.10 ± 0.30 c	7.28 ± 0.08 a	ND	ND	ND	17.76 ± 0.15 b	ND	1.12 ± 0.03 c	22.19 ± 0.19 d
Condensed tannins	ND	ND	ND	ND	ND	ND	ND	ND	ND	ND	ND	ND	ND	ND	ND	20.50 ± 0.18	ND	ND	ND	ND	ND	ND	ND	15.42 ± 0.14
Flavanones	31.77 ± 0.26 a	0.86 ± 0.02 b	ND	ND	2.06 ± 0.04 c	ND	2.96 ± 0.04 d	ND	14.24 ± 0.12 a	ND	ND	ND	11.59 ± 0.11 b	ND	10.84 ± 0.10 b	ND	ND	ND	ND	8.24 ± 0.08 a	27.30 ± 0.24 b	ND	0.37 ± 0.01 c	4.20 ± 0.06 d
Flavone glycosides	3.87 ± 0.05 a	0.08 ± 0.01 b	ND	0.22 ± 0.01 c	1.07 ± 0.02 d	0.22 ± 0.01 d	0.02 ± 0.01 e	ND	0.157 ± 0.01 a	0.30 ± 0.02 b	0.05 ± 0.01 c	0.09 ± 0.01 c	1.49 ± 0.03 d	3.86 ± 0.05 e	0.01 ± 0.00 f	ND	ND	ND	ND	2.44 ± 0.03 a	ND	ND	0.26 ± 0.01 b	ND
Flavonol glycosides	115.03 ± 0.30 a	7.98 ± 0.07 b	5.48 ± 0.06 b	17.17 ± 0.12 c	15.96 ± 0.11 c	1.52 ± 0.03 d	11.13 ± 0.10 e	1.30 ± 0.03 f	59.42 ± 0.23 a	0.42 ± 0.01 b	4.96 ± 0.05 c	4.66 ± 0.05 c	42.99 ± 0.18 d	0.05 ± 0.01 e	39.26 ± 0.19 d	0.30 ± 0.01 b	0.73 ± 0.01 a	0.88 ± 0.02 b	1.15 ± 0.02 c	1.05 ± 0.02 c	ND	3.09 ± 0.04 d	1.71 ± 0.03 e	0.88 ± 0.02 b
Flavonols	7.23 ± 0.07 a	13.21 ± 0.11 b	4.90 ± 0.05 c	38.65 ± 0.23 d	12.59 ± 0.11 b	3.86 ± 0.04 e	1.56 ± 0.03 f	4.31 ± 0.05 c	9.65 ± 0.08 a	0.90 ± 0.02 b	1.40 ± 0.03 c	7.40 ± 0.07 d	8.77 ± 0.08 d	1.04 ± 0.02 c	2.22 ± 0.04 e	0.20 ± 0.01 f	3.95 ± 0.05 a	1.93 ± 0.03 b	11.66 ± 0.10 c	4.25 ± 0.05 a	3.54 ± 0.04 a	12.59 ± 0.11 c	8.50 ± 0.08 e	1.99 ± 0.03 b
Flavones	6.67 ± 0.07 a	0.12 ± 0.01 b	ND	0.37 ± 0.01 c	1.44 ± 0.03 d	0.36 ± 0.01 e	0.03 ± 0.01 f	ND	0.26 ± 0.01 a	0.51 ± 0.01 b	0.08 ± 0.01 c	0.16 ± 0.01 a	2.42 ± 0.03 d	6.78 ± 0.07 e	0.03 ± 0.00 f	ND	ND	ND	ND	4.31 ± 0.05 a	ND	ND	0.43 ± 0.01 b	1.43 ± 0.02 c
Anthocyanins	10.24 ± 0.10 a	2.06 ± 0.04 b	0.44 ± 0.01 c	ND	1.78 ± 0.03 d	0.01 ± 0.00 e	0.25 ± 0.01 f	123.77 ± 0.45 g	1.18 ± 0.02 a	ND	ND	ND	8.81 ± 0.08 b	ND	0.90 ± 0.02 c	ND	0.40 ± 0.01 a	ND	3.15 ± 0.04 b	0.81 ± 0.02 c	0.86 ± 0.02 c	1.95 ± 0.03 d	0.08 ± 0.01 e	ND
Phenylpropanoid glycosides	ND	ND	ND	ND	ND	ND	ND	ND	ND	ND	ND	ND	ND	ND	ND	ND	ND	ND	ND	ND	ND	ND	ND	ND
Stilbenes	ND	ND	ND	ND	ND	ND	ND	ND	0.03 ± 0.00	ND	ND	ND	ND	ND	ND	ND	0.03 ± 0.01	ND	ND	ND	ND	ND	ND	ND
Lignans	0.71 ± 0.02 a	0.15 ± 0.01 b	ND	0.39 ± 0.01 c	1.37 ± 0.03 d	ND	0.26 ± 0.01 c	ND	1.25 ± 0.03 a	1.02 ± 0.02 a	8.12 ± 0.08 b	1.23 ± 0.03 a	2.11 ± 0.04 c	ND	0.04 ± 0.00 d	ND	0.60 ± 0.02 a	0.90 ± 0.02 b	0.31 ± 0.01 c	0.14 ± 0.01 d	0.19 ± 0.01 d	0.17 ± 0.01 d	0.14 ± 0.01 d	ND
Secoiridoids	115.17 ± 0.30 a	23.50 ± 0.21 b	ND	64.55 ± 0.26 c	72.90 ± 0.29 d	ND	42.41 ± 0.20 e	ND	104.86 ± 0.28 a	165.38 ± 0.35 b	465.87 ± 0.90 c	157.57 ± 0.33 d	244.70 ± 0.50 e	ND	5.94 ± 0.06 f	ND	38.22 ± 0.19 a	50.22 ± 0.22 b	50.33 ± 0.22 b	22.08 ± 0.16 c	30.06 ± 0.19 d	28.08 ± 0.18 d	22.11 ± 0.16 c	7.36 ± 0.07 e

Distinct lower-case letters indicate statistically significant differences (*p* < 0.05) between recipes belonging to the same country for the phenolic family concerned. F1: *Ratatouille*; F2: *Fish stew*; F3: *Cassoulet*; F4: *Basque pepper stew*; F5: *Meatballs with sauce*; F6: *French beef stew*; F7: *Tielle sétoise*; F8: *Basque cake*; I1: *Stewed dolphinfish*; I2: *Sardine meatballs*; I3: *Green olives pate*; I4: *Raw of sea bream and croaker fish with mango and green olives*; I5: *Sicilian caponata*; I6: *Bread and chickpea flour fritters*; I7: *Margherita pizza*; I8: *Tiramisu*; S1: *Garlic rabbit*; S2: *Valencian hake*; S3: *Potato omelette*; S4: *Fish suquet*; S5: *Valencian titaina*; S6: *Zarangollo*; S7: *Valencian paella*; S8: *Torrija with horchata; ND: not detected*.

#### 3.3.3. Mediterranean African Countries

The Mediterranean African countries (Algeria, Morocco, and Tunisia) showed intermediate phenolic levels between the European Mediterranean and non-Mediterranean regions, but with considerable variability among samples (Table 5). Phenolic acids remained the abundant family, with concentrations ranging from 11.39 mg/kg DW (in *Chicken tagine with olive and preserved lemons* (M4)) to 500.85 mg/kg DW (in *Kafteji* (T4)) across the three countries. Secoiridoids were consistently detected (up to 253.74 mg/kg DW in Algeria, 201.98 mg/kg DW in Morocco, and 124.94 mg/kg DW in Tunisia), while simple phenols were detected at lower contents with an average value of 9.13 mg/kd DW. This trend can reflect the use of olive-derived products and, thus, confirm the persistence of Mediterranean dietary patterns in North Africa. Flavonoids were present at moderate levels: flavonols (ranging from 0.48 to 36.98 mg/kg DW in the recipes T7: *Kamounia*, and T6: *Mloukhia*, respectively) and flavonol glycosides (up to 65.99 mg/kg DW in the *Mloukhia*) were the main subclasses, while flavan-3-ols and flavanones occasionally reached high contents (e.g., 51.38 and 19.66 mg/kg DW, respectively). Minor classes such as anthocyanins, flavones, and lignans were present in trace amounts (less than 5 mg/kg DW on average), while stilbenes were not detected across the recipes of the three countries. The remarkable presence of secoiridoids, phenolic acids, and simple phenols in these countries highlights a phytochemical composition similar to that of Southern Europe, shaped by shared agricultural and dietary traditions.

**Table 5:** Average contents (in mg/kg of DW) of phenolic compounds, expressed as the sum of each phenolic family, in recipes belonging to the Mediterranean African Countries under study. Distinct lower-case letters indicate statistically significant differences (*p* < 0.05) between recipes belonging to the same country for the phenolic family concerned.

	Algeria								Morocco								Tunisia							
Phenolic Family	A1	A2	A3	A4	A5	A6	A7	A8	M1	M2	M3	M4	M5	M6	M7	M8	T1	T2	T3	T4	T5	T6	T7	T8
Phenolic acids	141.06 ± 0.31 a	115.9 ± 0.52 b	91.55 ± 0.26 c	142.26 ± 0.6 a	13.26 ± 0.12 d	133.39 ± 0.28 e	39.31 ± 0.3 f	73.56 ± 0.2 g	58.04 ± 0.21 a	45.95 ± 0.18 b	66.63 ± 0.16 c	11.39 ± 0.11 d	76.69 ± 0.23 e	99.92 ± 0.25 f	28.51 ± 0.28 g	34.17 ± 0.13 h	95.78 ± 0.22 a	104.8 ± 0.59 b	76.61 ± 0.15 c	500.85 ± 0.41 d	86.17 ± 0.20 a	245.54 ± 0.30 e	29.04 ± 0.22 f	41.14 ± 0.2 g
Simple phenols	0.31 ± 0.01 a	0.37 ± 0.01 a	1.92 ± 0.03 b	1.54 ± 0.04 c	112.18 ± 0.24 d	3.22 ± 0.05 e	ND	ND	8.28 ± 0.14 a	5.19 ± 0.12 b	2.13 ± 0.04 c	23.01 ± 0.15 d	7.83 ± 0.06 a	3.44 ± 0.02 e	2.48 ± 0.02 c	7.93 ± 0.06 a	1.38 ± 0.03 a	0.95 ± 0.02 b	1.61 ± 0.04 a	0.87 ± 0.02 b	5.79 ± 0.11 c	0.65 ± 0.02 d	0.70 ± 0.02 d	ND
Flavan-3-ols	1.29 ± 0.02 a	4.59 ± 0.04 b	8.88 ± 0.05 c	14.27 ± 0.06 d	ND	1.70 ± 0.03 e	37.29 ± 0.1 f	ND	12.21 ± 0.12 a	20.15 ± 0.27 b	49.34 ± 0.23 c	ND	22.59 ± 0.19 b	ND	20.37 ± 0.27 b	30.12 ± 0.12 d	3.05 ± 0.03 a	38.63 ± 0.27 b	ND	ND	0.46 ± 0.02 c	ND	ND	51.38 ± 0.24 d
Condensed tannins	ND	ND	ND	ND	ND	ND	ND	ND	ND	2.95 ± 0.02 a	ND	ND	ND	ND	ND	20.58 ± 0.29 b	ND	10.54 ± 0.10 a	ND	ND	ND	ND	ND	32.14 ± 0.11 b
Flavanones	ND	ND	ND	0.92 ± 0.04 a	ND	ND	ND	8.48 ± 0.09 b	ND	1.21 ± 0.01	ND	ND	ND	ND	ND	ND	4.81 ± 0.05 a	4.86 ± 0.02 a	19.66 ± 0.06 b	7.02 ± 0.15 c	4.24 ± 0.03 a	1.36 ± 0.02 d	2.30 ± 0.03 e	ND
Flavone glycosides	0.62 ± 0.04 a	2.22 ± 0.04 b	4.43 ± 0.05 c	7.15 ± 0.11 d	1.03 ± 0.04 e	ND	ND	ND	0.94 ± 0.03 a	0.60 ± 0.02 b	ND	0.34 ± 0.01 c	5.13 ± 0.05 d	0.08 ± 0.02 e	0.48 ± 0.01 f	ND	ND	0.2 ± 0.01	ND	ND	0.22 ± 0.02	0.93 ± 0.02	ND	ND
Flavonol glycosides	0.14 ± 0.01 a	0.40 ± 0.02 b	0.37 ± 0.01 c	1.84 ± 0.02 d	0.56 ± 0.02 e	ND	3.89 ± 0.04 f	4.95 ± 0.04 g	ND	0.55 ± 0.05 a	3.81 ± 0.03 b	6.96 ± 0.07 c	0.37 ± 0.02 a	18.95 ± 0.12 d	4.26 ± 0.03 b	ND	2.92 ± 0.03 a	18.19 ± 0.09 b	7.72 ± 0.07 c	ND	16.41 ± 0.09 b	65.99 ± 0.14 d	8.33 ± 0.11 c	ND
Flavonols	0.84 ± 0.01 a	1.40 ± 0.04 b	1.87 ± 0.02 c	5.56 ± 0.08 d	1.46 ± 0.02 b	0.64 ± 0.04 a	17.24 ± 0.11 e	11.2 ± 0.07 f	1.44 ± 0.02 a	4.24 ± 0.01 b	16.41 ± 0.06 c	15.20 ± 0.08 c	4.96 ± 0.04 b	12.13 ± 0.06 d	14.79 ± 0.13 e	31.11 ± 0.12 f	11.13 ± 0.12 a	8.44 ± 0.08 b	2.25 ± 0.03 c	11.26 ± 0.07 a	3.19 ± 0.04 d	36.98 ± 0.25 e	0.48 ± 0.02 f	5.79 ± 0.12 g
Flavones	1.08 ± 0.01 a	3.83 ± 0.02 b	7.76 ± 0.07 c	12.51 ± 0.12 d	1.71 ± 0.04 e	ND	ND	ND	4.71 ± 0.04 a	1.11 ± 0.05 b	ND	0.66 ± 0.04 c	11.64 ± 0.09 d	0.15 ± 0.02 e	0.95 ± 0.02 b	5.93 ± 0.12 f	ND	0.34 ± 0.02 a	ND	ND	0.39 ± 0.01 a	1.61 ± 0.03 b	ND	1.77 ± 0.04 b
Anthocyanins	0.13 ± 0.01 a	ND	ND	0.03 ± 0.01 b	0.09 ± 0.01 c	0.15 ± 0.01 d	0.33 ± 0.01 e	0.27 ± 0.01 e	ND	0.12 ± 0.02 a	ND	ND	0.47 ± 0.01 b	0.63 ± 0.03 b	0.21 ± 0.01 c	ND	0.31 ± 0.02 a	0.40 ± 0.02 a	0.73 ± 0.04 b	3.93 ± 0.01 c	0.58 ± 0.01 b	1.34 ± 0.02 d	0.19 ± 0.01 e	ND
Phenylpropanoid glycosides	ND	ND	ND	ND	ND	ND	ND	ND	ND	ND	ND	ND	ND	ND	ND	ND	ND	ND	ND	ND	ND	ND	ND	ND
Stilbenes	ND	ND	ND	ND	ND	ND	ND	ND	ND	ND	ND	ND	ND	ND	ND	ND	ND	ND	ND	ND	ND	ND	ND	ND
Lignans	0.04 ± 0.01 a	0.05 ± 0.01 a	0.26 ± 0.01 b	0.21 ± 0.01 b	5.04 ± 0.06 c	0.44 ± 0.01 d	ND	ND	1.09 ± 0.02 a	0.69 ± 0.03 b	0.29 ± 0.01 c	1.76 ± 0.03 d	1.04 ± 0.02 a	0.47 ± 0.01 b	0.33 ± 0.01 e	ND	0.18 ± 0.01 a	0.12 ± 0.01 b	0.22 ± 0.02 a	0.12 ± 0.02 b	0.77 ± 0.03 c	0.09 ± 0.01 d	0.09 ± 0.01 d	ND
Secoiridoids	6.70 ± 0.13 a	7.92 ± 0.11 a	41.39 ± 0.24 b	33.24 ± 0.21 c	253.74 ± 0.25 d	69.49 ± 0.11 e	ND	ND	178.78 ± 0.22 a	111.92 ± 0.37 b	45.90 ± 0.18 c	201.98 ± 0.45 d	168.96 ± 0.55 e	74.14 ± 0.18 f	53.44 ± 0.27 g	9.08 ± 0.12 h	29.75 ± 0.29 a	20.45 ± 0.25 b	34.71 ± 0.29 c	18.81 ± 0.11 b	124.94 ± 0.22 d	13.96 ± 0.15 e	15.05 ± 0.10 e	ND

A1: *Rechta*; A2: *White chorba*; A3: *Mtewem*; A4: *Dobara*; A5: *Olive tagine*; A6: *Beans olive oil*; A7: *Sweet lamb*; A8: *Shakshuka*; M1: *Bissara*; M2: *Harira*; M3: *Chicken vermicelli*; M4: *Chicken tagine with olive and preserved lemons*; M5: *Lamb/beef tagine with peas and artichokes*; M6: *Tagine with quinces and honey*; M7: *Beef tagine with apricots, prunes and almonds*; M8: *Minichicken bastille*; T1: *Djerbian rice*; T2: *Tunisian lentil soup*; T3: *Ojja with merguez*; T4: *Kafteji*; T5: *Couscous with meat and vegetables*; T6: *Mloukhia*; T7: *Kamounia*; T8: *Assidat zgougou*; ND: not detected.

#### 3.3.4. Non-Mediterranean European Countries

Non-Mediterranean European countries (Germany and Luxembourg) exhibited lower levels of phenolic contents compared to Mediterranean regions (Table 6). The abundant class was phenolic acids, with values ranging from 36.91 to 324.60 mg/kg DW (in *Rice pudding with sugar and cinnamon* (G4) and *Pea sou *(G3), respectively), representing the major contributors to total phenolics. Flavonols and flavonol glycosides were detected in modest concentrations (average values of 8.00 and 9.16 mg/kg DW, respectively), whereas flavan-3-ols (up to 25.44 mg/kg DW in *Gulasch* (G1)) and flavones (as high as 7.96 mg/kg DW in *Judd with broad beans* (L1)) were present in smaller contents. Other families, such as simple phenols, flavonones, flavone glucosides, stilbenes, lignans, and secoiridoids, were either not detected or present at trace levels (less than 2 mg/kg DW). These data confirm that the phenolic profile of non-Mediterranean European countries is primarily defined by phenolic acids and simple flavonoids.

**Table 6:** Average contents (in mg/kg of DW) of phenolic compounds, expressed as the sum of each phenolic family, in recipes belonging to the non-Mediterranean European countries under study.

	Germany				Luxemburg			
Phenolic Family	G1	G2	G3	G4	L1	L2	L3	L4
Phenolic acids	68.95 ± 0.42 a	120.76 ± 0.63 b	324.60 ± 1.15 c	36.91 ± 0.27 d	77.48 ± 0.48 a	244.77 ± 0.92 b	120.27 ± 0.61 c	72.23 ± 0.45 a
Simple phenols	ND	ND	ND	ND	ND	ND	ND	ND
Flavan-3-ols	25.44 ± 0.18 a	ND	14.36 ± 0.11 b	18.68 ± 0.15 c	8.84 ± 0.09 a	4.45 ± 0.05 b	ND	2.90 ± 0.04 c
Condensed tannins	ND	ND	ND	18.22 ± 0.14	ND	ND	ND	ND
Flavanones	1.59 ± 0.03 a	ND	ND	0.46 ± 0.01 b	ND	ND	ND	ND
Flavone glycosides	0.20 ± 0.01 a	ND	0.06 ± 0.01 b	ND	1.92 ± 0.03	ND	ND	ND
Flavonol glycosides	9.30 ± 0.07 a	57.45 ± 0.28 b	0.98 ± 0.02 c	0.91 ± 0.02 c	2.09 ± 0.03 a	1.45 ± 0.02 b	0.71 ± 0.01 c	0.36 ± 0.01 d
Flavonols	11.65 ± 0.09 a	18.27 ± 0.14 b	11.72 ± 0.09 c	2.32 ± 0.03 d	6.33 ± 0.06 a	10.71 ± 0.09 b	1.73 ± 0.02 c	1.24 ± 0.02 c
Flavones	0.33 ± 0.01 a	ND	0.11 ± 0.01 b	0.92 ± 0.02 c	7.96 ± 0.07	ND	ND	ND
Anthocyanins	0.48 ± 0.01 a	ND	2.64 ± 0.04 b	ND	ND	1.95 ± 0.03 a	0.38 ± 0.01 b	0.06 ± 0.01 c
Phenylpropanoid glycosides	ND	ND	ND	ND	ND	ND	ND	ND
Stilbenes	0.29 ± 0.01	ND	ND	ND	ND	ND	ND	ND
Lignans	ND	ND	ND	ND	ND	ND	ND	ND
Secoiridoids	ND	ND	ND	ND	ND	ND	ND	ND

Distinct lower-case letters indicate statistically significant differences (*p* < 0.05) between recipes belonging to the same country for the phenolic family concerned. G1: *Gulasch*; G2: *Königsberger meatballsx*; G3: *Pea soup*; G4: *Rice pudding with sugar and cinnamon*; L1: *Judd with broad beans*; L2: *Green bean soup*; L3: *Plum tart*; L4: *Tarte tatin with vanilla ice cream*; ND: not detected.

**Figure 9:**
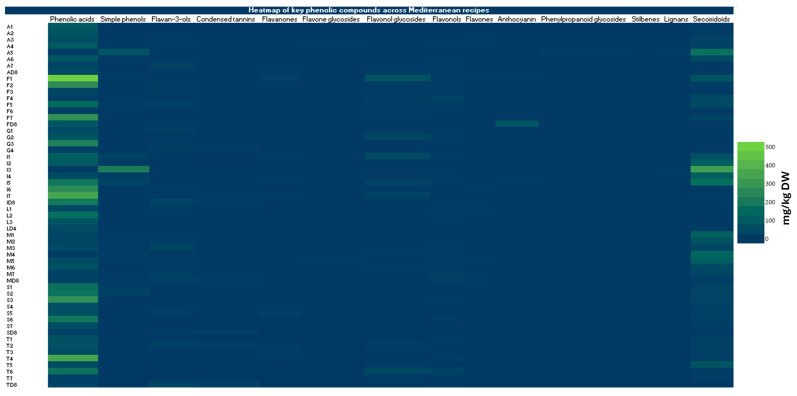
Heatmap of the distribution of key phenolic families across Mediterranean and non-Mediterranean recipes.

For a better comparison of the three regional groups, a heatmap was conducted to illustrate the distribution and relative abundance of the main phenolic families across the groups (Figure 9). The color intensity, ranging from dark blue (low or non-detectable) to bright green (high concentration), provides an overview of the compositional variability within and between the three groups.

As illustrated in Figure 9, a clear regional clustering pattern can be noticed. European Mediterranean samples exhibited the most intense green coloration, particularly for phenolic acids and secoiridoids, confirming their abundance in recipes typical of Southern Europe. Moderate intensities are also visible for flavonols, flavonol glycosides, and flavanones, suggesting a diversified phenolic composition. Mediterranean African samples displayed a quite similar but slightly less intense pattern. In more detail, the heatmap indicates moderate levels of phenolic acids and secoiridoids, with occasional enrichment in flavonol glycosides and flavan-3-ols. However, the overall phenolic abundance appears less intense when compared with the European Mediterranean group. In contrast, non-Mediterranean European recipes (bottom of the heatmap) showed limited green intensity, indicating generally lower phenolic levels. The abundance of phenolic acids with minor to no secoiridoids or lignans highlights a less diverse phenolic composition, typical of diets with reduced olive oil consumption.

Hence, the heatmap visually reinforces the quantitative results presented in Table 4, Table 5 and Table 6 and Tables S1–S3 in the Supplementary Material: European Mediterranean recipes seem to contain the richest and most diversified phenolic profiles, followed by Mediterranean African ones, while non-Mediterranean European recipes were linked to a simpler, less phenolic-rich composition. The prominence of phenolic acids and secoiridoids across Mediterranean groups provides clear evidence of the shared nutritional and functional identity of the Mediterranean dietary pattern.

### 3.4. Statistical Multivariate Analysis

#### 3.4.1. Association Patterns Between Polyphenol Measures and Antioxidant Capacity Using Simple Linear Regression Models

Simple linear regression analyses (Table 7) indicated differing association patterns between polyphenol measures and antioxidant capacity depending on the assay used. For TEAC, total polyphenol content (TP_total) was not significantly associated with antioxidant capacity (R^2^ = 0.007, *p* = 0.533). In contrast, when extractable polyphenols were examined (Model 2), TP_ext showed a statistically significant positive association with TEAC (b = 11.96, *p* = 0.031), accounting for a modest proportion of variance (R^2^ = 0.156).

The inclusion of standardized enrichment indicators for flavonoids and phenolic acids did not improve model fit, and neither variable was independently associated with TEAC. This suggests that variation in TEAC is more closely related to extractable polyphenol content than to differences in phenolic subclass enrichment. In the fraction model (Model 3), TP_ext remained a significant positive predictor of TEAC, whereas non-extractable polyphenols (TP_non_ext) were not associated with antioxidant capacity, supporting a more evident statistical association between TEAC and extractable polyphenols.

For ORAC, none of the simple linear models identified statistically significant associations with total or fractionated polyphenol measures. TP_total, TP_ext, flavonoid enrichment, phenolic acid enrichment, and TP_non_ext were all non-significant predictors (all *p* > 0.32), and explained variance was consistently low (R^2^ ≤ 0.065). These findings indicate that, in contrast to TEAC, ORAC values were not detectably associated with polyphenol quantity or fractionation in these recipes.

**Table 7:** Linear regression models examining associations between polyphenol content, composition, and total antioxidant capacity (TEAC) in traditional Mediterranean recipes.

		UC		SC	t	sig	R	SSE	R^2^	Adj. R^2^	ANOVA	
		b	SE	β							F	*p*-Value
	TEAC											
Model 1	(Constant)	5335.22	586.88		9.09	<0.001		2529.04	0.007	−0.011	0.39	0.533
	TP_total	−0.32	0.51	−0.09	−0.63	0.533	0.085					
Model 2	(Constant)	3167.707	725.480		4.366	<0.001		2376.82	0.156	0.107	3.20	0.031
	TP.Extr	11.958	4.179	0.410	2.861	0.006	0.394					
	z.Flav.Extr	−128.659	362.841	−0.051	−0.355	0.724						
	z.Phenolic.Acid.Extr	27.861	345.228	0.011	0.081	0.936						
Model 3	(Constant)	3695.267	727.746		5.078	<0.001	0.425	2319.30	0.181	0.15	5.84	0.005
	TP.Extr	11.749	3.634	0.403	3.233	0.002						
	TP.non.Extr	−0.623	0.472	−0.165	−1.321	0.192						
ORAC												
Model 1	(Constant)	66,285.851	6954.610		9.53	<0.001		29,969.46316	0.009	−0.009	0.50	0.481
	TP_total	−4.244	5.983	−0.096	−0.709	0.481	0.096					
Model 2	(Constant)	68,814.601	9057.984		7.597	<0.001		29,675.88	0.065	0.01	1.20	0.321
	TP.Extr	−42.041	52.176	−0.122	−0.806	0.424	0.254					
	z.Flav.Extr	8466.576	4530.246	0.284	1.869	0.067						
	z.Phenolic.Acid.Extr	−544.164	4310.339	−0.018	−0.126	0.900						
Model 3	(Constant)	65,745.627	9491.437		6.927	<0.001	0.097	30,248.83	0.009	−0.028	0.251	0.779
	TP.Extr	−0.270	47.391	−0.001	−0.006	0.995						
	TP.non.Extr	−4.345	6.156	−0.097	−0.706	0.483						

UC = unstandardized coefficient; SE = standard error; SC = standardized coefficient (β); t = t-value; sig = *p*-value; R = correlation coefficient; SSE = standard error of the estimate; R^2^ = coefficient of determination; Adj. R^2^ = adjusted R^2^; ANOVA = analysis of variance; F = F-statistic. N = 56. All VIF ≤ 1.3, Residual statistics: no extreme leverage or Cook’s distance issues.

#### 3.4.2. Association Patterns Between Polyphenol Measures and Antioxidant Capacity with Country-Level Clustering

When country-level clustering was accounted for using linear mixed-effects models with random intercepts (Table 8), fixed-effect estimates were broadly comparable to those obtained from simple regression analyses, while substantial between-country variability was evident for both antioxidant assays. For TEAC, TP_total was not associated with antioxidant capacity (*p* = 0.533), whereas TP_ext remained significantly and positively associated with TEAC in both the composition (Model 2) and fraction (Model 3) models (*p* ≤ 0.006). Standardized enrichment indicators for flavonoids and phenolic acids did not explain additional variability beyond TP_ext. Similarly, in the fraction model, only extractable polyphenols were independently associated with TEAC, whereas non-extractable polyphenols were not.

Across all TEAC mixed-effects models, the intraclass correlation coefficient (ICC ≈ 0.33) indicated that approximately one-third of the total variance was attributable to differences between countries. Marginal R^2^ values (0.005–0.125) showed that fixed effects alone explained a limited proportion of variance, whereas conditional R^2^ values (0.333–0.413) indicated that incorporating country-level random effects substantially improved overall model fit.

For ORAC, none of the fixed-effect predictors reached statistical significance in any mixed-effects model. Total, extractable, and non-extractable polyphenol content were all unrelated to ORAC values (all *p* ≥ 0.42). Nevertheless, ICC estimates again approached 0.33, and conditional R^2^ values (~0.33–0.36) indicated that a substantial proportion of ORAC variability was attributable to between-country differences, despite minimal variance explained by the polyphenol predictors themselves.

**Table 8:** Linear mixed-effects models accounting for country-level clustering in the association between polyphenol content, composition, and TEAC/ORAC in traditional Mediterranean recipes.

		Fixed Effects					Random Effects and Model Fit				
		b	SE	t	sig	95% CI	ICC	Marginal R^2^	Conditional R^2^	−2LL	AIC
TEAC											
Model 1	(Intercept)	5335.2	1867.0	2.86	0.006	[1592.3–9078.1]	0.33	0.005	0.333	1020.55	1024.55
	TP_total	−0.32	0.51	−0.63	0.533	[−1.33–0.70]					
Model 2	(Intercept)	3167.71	1816.73	1.744	0.087	[−477.8–6813.2]	0.33	0.105	0.400	980.63	984.63
	TP.Extr	11.958	4.179	2.862	0.006	[3.57–20.34]					
	z.Flav.Extr	−128.659	362.830	−0.355	0.724	[−856.7–599.4]					
	z.Phenolic.Acid.Extr	27.861	345.218	0.081	0.936	[−664.9–720.6]					
Model 3	(Intercept)	3695.267	1780.753	2.075	0.043	[123.53–7267.01]	0.33	0.125	0.413	1005.75	1009.75
	TP.Extr	11.749	3.633	3.234	0.002	[4.46–19.04]					
	TP.non.Extr	−0.623	0.472	−1.321	0.192	[−1.57–0.32]					
ORAC											
Model 1	(Intercept)	66,285.851	22,123.084	54	2.996	[21,931.7–110,639.9]	0.33	0.006	0.333	1287.57	1291.57
	TP_total	−4.244	5.983	54	−0.709	[−16.24–7.75]					
Model 2	(Intercept)	68,814.601	22,682.795	3.034	0.004	[23,298.24–114,330.96]	0.33	0.042	0.357	1243.19	1247.19
	TP.Extr	−42.041	52.175	−0.806	0.424	[−146.73–62.66]					
	z.Flav.Extr	8466.576	4530.117	1.869	0.067	[−623.77–17,556.920]					
	z.Phenolic.Acid.Extr	−544.164	4310.215	−0.126	0.900	[−9193.24–8104.92]					
Model 3	(Intercept)	65,745.627	23,225.008	2.831	0.007	[19,162.16–112,329.10]	0.33	0.006	0.333	1277.98	1281.98
	TP.Extr	−0.270	47.387	−0.006	0.995	[−95.32–94.78]					
	TP.non.Extr	−4.345	6.156	−0.706	0.483	[−16.69–8.001]					

B = unstandardized regression coefficient; SE = standard error; CI = confidence interval; ICC = intraclass correlation coefficient; −2LL = −2 restricted log likelihood; AIC = Akaike information criterion. Random intercept: Country; Number of recipes (N)= 56; Number of countries= 8.

## 4. Discussion

### 4.1. Total Antioxidant Capacity and Polyphenolic Profile

#### 4.1.1. Mediterranean European Countries

The high values of TAC from France, Spain, and Italy can be ascribed to the combination of ingredients rich in polyphenols and bioactive compounds, including vegetables, legumes, aromatic herbs, and extra virgin olive oil (EVOO) [53,54,55]. The highest TAC in this group is for *valencian hake *(S2) (TEAC methodology), a fact that can be attributed to the combination of EVOO, onion, and black pepper, ingredients rich in phenolic compounds with recognized antioxidant capacity [54,56]. The presence of phenolic acids, simple phenols, and secoiridoids in high contents within this group can be attributed to the use of EVOO, which is known to be particularly rich in compounds belonging to these phenolic families, mainly hydroxytyrosol, tyrosol, and oleuropein derivatives [57]. Additionally, fish, particularly lean species such as hake, provides bioactive peptides with antioxidant properties, contributing to the overall TAC of the dish [55,58,59]. Another similar recipe, *gilthead sea bream in salt with grilled vegetables* [31], shows a combination of similar ingredients, optimizing the antioxidant potential of the dish.

Similarly, in the case of *fish stew* (F2), its high ORAC in TAC can be explained by the synergy between the omega-3 fatty acids from fish and seafood and the polyphenols present in spices such as saffron, paprika, and garlic, which have been shown to enhance the antioxidant capacity of dishes in which they are included [60,61,62]. Likewise, *sardine meatballs* (I2) and *cod meatball stews* [31] display high antioxidant responses, likely due to the combination of marine proteins, healthy fats, and vegetable-based sauces such as tomato. These preparations highlight the antioxidant potential of marine proteins combined with vegetable ingredients and spices, a characteristic pattern in Mediterranean recipes.

Recipes such as *cassoulet* (F3), *fish suquet* (S4), and *gypsy pot* [31] stand out for incorporating legumes, vegetables, and sometimes marine proteins. These Mediterranean dishes combine vegetables and animal-based foods, illustrating how distinct culinary traditions converge to create dishes with notable antioxidant potential.

On the other hand, recipes with lower TAC, such as *tielle sétoise* (F7) (TEAC) and *potato omelette* (S3) (ORAC), reflect the negative impact of using ingredients with low antioxidant activity, such as flour, butter, and eggs. Although eggs contain carotenoids, such as lutein and zeaxanthin, their contribution is limited compared to plant-based sources richer in polyphenols [56]. These findings suggest that the antioxidant potential of a dish is not strictly determined by the concentration of polyphenols but also by potential synergistic or antagonistic interactions among ingredients, which may either enhance or inhibit the effective antioxidant activity of individual phenolic compounds [63,64] Furthermore, butter, being an animal fat, has a low antioxidant capacity [65], and the use of refined flours implies the removal of bioactive compounds found in whole grains [66].

Italian recipes generally show a lower diversity of plant-based ingredients compared to those from France and Spain, which partly explains their more moderate phenolic content. However, dishes such as *margherita pizza *(I7) and *raw sea bream with croaker fish, mango, and green olives* (I4) feature high TAC values due to the combination of fruits, vegetables, and EVOO [55,58,59]. In contrast, recipes such as *green olives paté* (I3) show moderate values since olives, though a source of polyphenols, are not complemented by other antioxidant-rich ingredients.

Finally, *Valencian paella* (S7) and *rice with vegetables and cod* [31] both integrate cereals, vegetables, and fish, unified by the use of EVOO as the main culinary fat, contributing to a balanced antioxidant profile that reflects both phenolic content and ORAC activity.

In general, selected recipes from France and Spain follow a similar pattern with a predominance of vegetables, legumes, and spices. This is different from Italy, which has a lower diversity of plant-based ingredients in comparison.

These results align with findings from Slovenian preschool menus, where the inclusion of antioxidant-rich foods such as fruits, vegetables, whole grains, and nuts led to significantly higher TAC values and improved nutritional profiles, particularly when assessed via ABTS and FRAP assays. This supports the idea that ingredient diversity and fiber content play a key role in enhancing the antioxidant potential of complete meals [67].

#### 4.1.2. Mediterranean African Countries

The culinary pattern in this region is characterized by the abundant use of spices, legumes, and vegetables, which explains the high TAC values in recipes such as *rechta* (A1), *chicken tagine with olives and preserved lemons* (M4), and *harira *(M2). The ingredients that make up these recipes have been widely studied for their antioxidant capacity and their contribution of polyphenols [68]. Specifically, Tunisian recipes such as *mloukhia* (T6), *kamounia* (T7), and *kafteji* (T4) have the highest TEAC values. These findings align with previous studies that have reported high polyphenol content in spices such as cumin, garlic, paprika, and black pepper. Additionally, these studies highlight the high polyphenol content in vegetables such as bell pepper, tomato, and onion. These ingredients are essential in Maghreb cuisine [56].

The exceptional ORAC value of *mini chicken bastilla *(M8) (111,101.7 µmol Trolox/100 g DW) is due to its composition rich in nuts and spices, ingredients widely studied for their high antioxidant capacity [36,62]. It has been shown that almonds and other seeds containg high concentrations of flavonoids and phenolic acids, which significantly contribute to the TAC of the dishes in which they are included [56].

In contrast, recipes such as *chicken tagine with olives and preserved lemons *(M4) and *beef tagine with apricots, prunes, and almonds* (M7) present lower values in TEAC TAC. This observation could be explained by a lower density of polyphenol-rich ingredients or by culinary techniques such as prolonged cooking, which may degrade certain bioactive compounds [69]. Similary in ORAC TAC, recipes such as *tunisian lentil soup* (T2), *couscous with meat and vegetables* (T5), and *assidat zgougou* (T8) show reduced values. Probably the presence of legums and nuts, due to their complex food matrix, does not favor a high extraction of antioxidants [69]. However, since both recipes were found to exhibit quite rich and diversified phenolic profiles, these culinary practices may also be responsible for an increase in the antagonistic or inhibitory interactions between phenolic and non-phenolic compounds, thus inhibiting their antioxidant potential [70].

#### 4.1.3. Non-Mediterranean European Countries

In comparison to Mediterranean recipes, dishes from Germany and Luxembourg have considerably lower TP values and individual polyphenols contents, which can be explained by a lower presence of plant-based ingredients. In *gulasch* (G1) and *judd with broad beans* (L1), although spices and legumes are included, the base of the recipe is dominated by meat and sausages, wich limits their polyphenols content [53,55,66].

In addition, *rice pudding with sugar and cinnamon* (G4) and *tarte tatin with vanilla ice cream* (L4) show reduced values in TP due to their composition based on flour, sugar, egg, and butter. Although the cinnamon used in the German recipe is a source of polyphenols, its presence in the recipe is not sufficient to significantly increase the TAC [71,72]. Similarly, in the recipe from Luxemburg, the caramelization process may generate certain antioxidant compounds, but these do not compensate for the low polyphenol density of the main ingredients [73].

On the other hand, German recipes exhibit higher TAC values and polyphenols contents compared to Luxembourgish recipes, which may be due to the inclusion of ingredients rich in phenolic compounds, such as cabbage, potatoes, mustard, and marinated meats. Additionally, the use of prolonged cooking processes or fermentation may enhance the release of antioxidants [68].

In Luxembourg, although the phenolic contents were richer (notably phenolic acids) compared to other recipes, the lower TAC values in recipes such as *green bean soup* (L2) and *plum tart *(L3) could be attributed to the use of ingredients with lower antioxidant capacity and milder cooking methods, which can affect the potential activity of the recipe.

The observed differences between TEAC and ORAC can be explained by the varying sensitivity of each method to certain types of antioxidants. In this context, the high ORAC value of *tarte tatin with vanilla ice cream* (L4) suggests that part of its antioxidant capacity may be related to the formation of compounds during baking, such as Maillard reaction products [68]. Similarly, the high values in *königsberger meatballs* (G2) may be attributed to the presence of capers, which contain flavonoids such as quercentin and kaempferol, as well as the contribution of lemon juice, a source of vitamin C [74].

Similar trends were observed in hospital meals in Sweden and Bolivia, where Swedish dishes, richer in plant-based ingredients and fiber, showed higher TAC (FRAP, TEAC, and TP) compared to Bolivian meals. These differences were partly attributed to both the selection of ingredients and cooking techniques that preserve or reduce antioxidant compounds [75].

### 4.2. Extractable and Non-Extractable Antioxidant

The results obtained show that, for all the recipes in all the regions studied, the non-extractable fraction represents the largest percentage of the TAC. This finding aligns with previous studies that highlight the relevance of phenolic compounds bound to the food matrix, which are not available in conventional soluble extracts. In general, the non-extractable fraction exceeded 80% of TAC across all methods. However, differences were observed depending on the assay used. ORAC showed the most uniform pattern, with non-extractable contributions consistently around 90% in the vast majority of recipes, with only a few exceptions. In contrast, TEAC and TP displayed greater inter-recipe variability, with non-extractable values ranging from approximately 50% to 90% depending on the recipe [29,38,41,76].

Generally, the distribution of both fractions is influenced by both the composition of the ingredients and the culinary techniques employed. Recipes with a higher content of vegetables and spices present a greater proportion of extractable antioxidants due to their richness in water-soluble phenolic compounds, such as flavonoids and phenolic acids. In contrast, those based on cereals, legumes, nuts, and animal proteins tend to retain most of the antioxidants in the non-extractable fraction, due to their interaction with fibers, resistant starches, and proteins, which limit their solubility [10,68,77].

This study highlights the importance of considering both extractable and non-extractable fractions when evaluating the antioxidant capacity of traditional recipes, in line with previous research such as that by Durazzo et al. [29], who also emphasized the relevance of the non-extractable fraction. Unlike the latter, this study analyzed a greater variety of Mediterranean recipes and used different methods, such as ORAC, TEAC, and TP, which explains some of the differences in the results. In addition, a detailed analysis of the phenolic compound profile was carried out. Additionally, it aligns with the findings of Pérez-Jiménez et al. [39], who warned that conventional methods underestimate antioxidant capacity by not considering the non-extractable fraction, which can be released during digestion and provide health benefits. The variability in the distribution of this fraction across different recipes suggests that its contribution to health may be greater than initially estimated.

#### 4.2.1. Mediterranean European Countries

The traditional recipes from France, Italy, and Spain follow this pattern. In France, *ratatouille* (F1), which includes tomato, bell pepper, and eggplant, presents the highest proportion of extractable antioxidants, especially in TEAC and TP (46% and 40%, respectively). A similar behavior is observed in Italy with *Sicilian caponata* (I5) and in Spain with *zarangollo* (S6), both rich in vegetables.

In contrast, recipes with a high proportion of proteins and complex carbohydrates or those subjected to intensive cooking techniques present very high values in the non-extractable fraction. Examples include *french beef stew* (F6) in France, *bread and chickpea flour fritters* (I6) in Italy, and *fish suquet* (S4) in Spain, which reach up to 95%.

Similarly, in the study by Durazzo et al. [28,29], on traditional Italian dishes such as *pasta alla carbonara* or *pasta alla amatriciana*, it was observed that the non-extractable fraction accounted for up to 97% of the total antioxidant content, confirming that complex and cooked matrices, especially those containing components such as cereals and animal fats, concentrate much of their antioxidant capacity in the non-extractable fraction.

In line with these findings, Cuenca-Ortolá et al. [30] also demonstrated that dishes from the Spanish MD contain a high proportion of non-extractable polyphenols, with contributions that can exceed 80%, especially in recipes with whole grains or legumes.

#### 4.2.2. Mediterranean African Countries

In Algeria, Morocco, and Tunisia, the same pattern is observed. In this case, recipes with a higher proportion of vegetables and spices, such as *white chorba* (A2) in Algeria, *beef with apricots, prunes, and almonds* (M7) in Morocco, and *assidat zgougou* (T8) in Tunisia, present a relatively high extractable fraction within each country (up to 32% in TEAC for the Tunisian case).

In contrast, dishes richer in cereals, legumes, and nuts show predominantly higher values in the non-extractable fraction. Examples include *rechta* (A1) in Algeria, *mini chicken bastilla* (M8) in Morocco, and *kefteji* (T4) in Tunisia, with values reaching up to 98–99% in some evaluation methods [68].

The results indicate that, in the traditional recipes of Algeria, Morocco, and Tunisia, the non-extractable fraction dominates the TAC. This fact is explained since food matrices rich in fibers, resistant starches, and proteins hinder the release of bioactive compounds, as they become trapped within the food structure and are not easily extracted with conventional solvents [10,77].

This lower plant diversity and higher proportion of ingredients of animal origin is associated, as has also been seen in Bolivian hospital dishes [75] or in Italian *besciamella* [29], with a lower TAC in free extracts, but a notable contribution from the bound polyphenols present in the fat and protein matrix, especially after heat treatments such as cooking or frying.

#### 4.2.3. Non-Mediterranean European Countries

In Germany and Luxembourg, the same trend is observed. Recipes that include vegetables and spices, such as *gulasch* (G1) in Germany and *green bean soup* (L2) in Luxembourg, present the highest proportions of extractable antioxidants, reaching up to 49% in TEAC for the German recipe.

On the other hand, recipes rich in cereals and starches show a predominant non-extractable fraction. *Pea soup* (G3) and *rice pudding with sugar and cinnamon* (G4) in Germany have values exceeding 87%, while *judd with broad beans* (L1) in Luxembourg reaches 93% in ORAC and 91% in TP. Both the presence of insoluble fiber and the structure of polyphenols in legumes promote greater retention of antioxidants within the food matrix. This is due to their high degree of polymerization and the strong interactions they establish with cell wall components, such as polysaccharides and proteins, which hinder their release and extraction by conventional methods [78,79].

A particular case is *tarte tatin with vanilla ice cream* (L4), which presents one of the highest non-extractable fractions (92% in ORAC). This suggests that part of its TAC comes from compounds formed during Maillard reactions and caramelization during cooking, which are not easily released with conventional extraction methods [10,68,77].

### 4.3. Comparison of Simple and Mixed Models: Role of Country-Level Clustering

Accounting for country-level clustering did not materially change the direction or statistical significance of fixed-effect associations but substantially altered the interpretation of variance structure. For TEAC, the positive association with extractable polyphenols was consistent across simple and mixed-effects models, suggesting a robust relationship within the constraints of the observational design. This aligns with established literature indicating that the TEAC assay, based on an ET mechanism, correlates strongly with the structural features of phenolic compounds [80,81,82]. Recent studies confirm that TEAC remains one of the most reliable indicators for phenolic content due to its high reproducibility and direct correlation with ET mechanisms [82]. However, the mixed-effects analyses additionally revealed that a considerable proportion of TEAC variability is structured at the country level, indicating meaningful cross-cultural or culinary heterogeneity.

For ORAC, the contrast was more pronounced. While simple linear models already showed no evidence of association with polyphenol measures, mixed-effects models further demonstrated that ORAC variability is largely driven by between-country differences rather than by polyphenol quantity or fractionation. This discrepancy is increasingly attributed to the high sensitivity of the ORAC assay to non-phenolic components and the specific food matrix, which can provide a more comprehensive, albeit more variable, assessment of antioxidant capacity in complex food mixtures compared to the TEAC assay [82,83]. The consistently high ICC values and low marginal R^2^ estimates suggest that factors related to recipe matrix, preparation practices, or non-phenolic antioxidants may contribute more strongly to ORAC values than polyphenol content alone. These methodological differences between assays have relevant implications for data interpretation. TEAC predominantly reflects the overall electron-donating capacity of antioxidant compounds present in the matrix, while ORAC evaluates the ability to scavenge peroxyl radicals across a broader range of antioxidant species acting through hydrogen atom transfer mechanisms. Consequently, these assays should be regarded as complementary rather than interchangeable, and their combined application provides a more comprehensive assessment of the total antioxidant capacity of compositionally complex foods [4,81].

Overall, these results indicate that TEAC is more closely associated with extractable polyphenol quantity, whereas ORAC appears to reflect broader, country-specific characteristics of traditional recipes. These findings highlight the importance of accounting for cultural and compositional context when interpreting antioxidant capacity measures and caution against assuming equivalence between different antioxidant assays. Moreover, the high antioxidant capacity of traditional Mediterranean recipes, driven predominantly by the non-extractable fraction, suggests that conventional nutritional databases likely underestimate their true bioactive potential, with relevant implications for dietary antioxidant intake and functional food research [83,84]. In fact, consuming a habitual diet high in antioxidants has been associated with decreased risk of death from all-causes, cancer, and cardiovascular disease [85,86].

The acid hydrolysis conditions applied for the extraction of the non-extractable fraction are aggressive, which may promote partial degradation of structural components of the matrix and the generation of secondary compounds liable to interfere with the analytical assays, especially in ORAC, potentially contributing to an overestimation of the determined values [87,88].

Several methodological considerations should be acknowledged when interpreting the present regression findings. First, although linear mixed-effects models were employed to account for potential clustering of recipes within countries, the number of observations per country was relatively limited. As a result, estimates of random-effect variance should be interpreted with caution, and the mixed-effects analyses were primarily intended as sensitivity analyses to evaluate the robustness of fixed-effect associations rather than to support strong country-specific inferences. The overall sample size and the uneven distribution of recipes across countries may have constrained statistical power, particularly for detecting small or moderate effects and for reliably estimating random-effects parameters. In addition, the relatively modest number of observations may affect model stability in multiple regression and mixed-effects analyses. Although parsimonious model specifications were adopted to reduce the risk of overfitting, smaller associations may not have been detectable, and variance components should be interpreted cautiously. Accordingly, the regression results should be viewed as indicative of general patterns within the dataset rather than as precise quantitative estimates. In addition, the relatively modest number of observations may affect model stability in multiple regression and mixed-effects analyses. Although parsimonious model specifications were adopted to reduce the risk of overfitting, smaller associations may not have been detectable, and variance components should be interpreted cautiously. Accordingly, the regression results should be viewed as indicative of general patterns within the dataset rather than as precise quantitative estimates.

Additionally, the Folin–Ciocalteu method measures overall reducing capacity through an ET-based mechanism rather than radical-scavenging activity directly and may respond to non-phenolic reducing compounds. Accordingly, TP values should be interpreted as complementary to, but distinct from, the TEAC and ORAC measures, which evaluate radical-scavenging [34].

## 5. Conclusions

This study provides a comprehensive view of the TAC in traditional MD recipes by considering both the extractable and non-extractable fractions using TEAC, ORAC, and TP methods and also supplying data on their phenolic profile.

The comparative analysis between regions shows that traditional recipes from Mediterranean countries (both European and African) present a greater diversity of antioxidant sources, attributed to the abundance of plant-based ingredients such as vegetables, legumes, spices, and EVOO. In these recipes, the extractable fraction is more prominent in those that include a higher proportion of these ingredients, although it is always surpassed by the non-extractable fraction, which predominates in dishes with cereals, nuts, and legumes.

On the other hand, in non-Mediterranean European countries, a lower TAC is observed, associated with a lower diversity of plant-based ingredients and a greater use of refined flours, meat products, and cooking methods such as baking and caramelization, which may favor the retention of non-extractable antioxidants.

The comparative assessment of phenolic profiles across the three regional groups revealed distinct trends aligned with their dietary patterns. The recipes of the European Mediterranean countries exhibited the richest and most diverse polyphenol composition, dominated mainly by phenolic acids, flavonoids, and secoiridoids, the latter reflecting the central role of olive oil in the MD. Meanwhile, recipes belonging to the non-Mediterranean European countries showed a simpler phenolic profile, characterized mainly by phenolic acids and minor flavonoid subclasses, consistent with diets less dependent on olive oil and plant-based ingredients typical of the Mediterranean basin. Mediterranean African countries were associated with intermediate phenolic profiles in their dishes, sharing with Southern Europe the predominance of phenolic acids and secoiridoids, though with slightly lower contents, reflecting both shared agricultural traditions and regional variations in ingredient use and culinary practices.

The heatmap analysis supported these trends, highlighting clear regional clustering and the prevalence of phenolic acids and secoiridoids in Mediterranean recipes, in contrast to the limited diversity observed in non-Mediterranean European dishes.

Unlike previous approaches focused solely on the soluble fraction, the results confirm that the non-extractable fraction represents much of the TAC, reaching values greater than 80%. This highlights the importance of considering both fractions to obtain a more complete assessment of the antioxidant potential of foods. In this context, given that the non-extractable fraction can be released during digestion and interact with the intestinal microbiota, its consideration in the evaluation of the bioactive potential of traditional Mediterranean recipes is especially relevant, both for more accurately estimating the actual dietary intake of antioxidant compounds and for supporting the development of functional foods based on these culinary patterns.

Regarding the comparison of simple and mixed-effects models, null or weak associations, especially in the latter, should not be interpreted as definitive evidence of abscence of effect. Future studies with larger and more balanced country-level samples would be valuable to further clarify the role of country- or cuisine-specific factors in determining antioxidant capacity.

These findings reinforce the significance of the MD as a model with high antioxidant potential and emphasize the relevance of considering the non-extractable fraction in recipe evaluations. Additionally, the results obtained can serve as a reference for future research on the stability and functionality of antioxidants in different combinations and culinary preparations and as a sort of database for nutritional intervention studies aiming at boosting dietary TAC for health improvement.

## Data Availability

The original contributions presented in this study are included in the article/Supplementary Material. Further inquiries can be directed to the corresponding authors.

## Supplementary Materials

The following supporting information can be downloaded at: https://www.mdpi.com/article/10.3390/antiox15030377/s1.

## Abbreviations

The following abbreviations are used in this manuscript:

AAPH	2,2-azinobis (2-amidinopropane) dihydrochloride
ABTS	2,2′-azinobis (3-ethylbenzothiazoline-6-sulfonic acid)
BPC	Base Peak Chromatogram
DW	Dry Weight
ET	Electron Transfer
EVOO	Extra Virgin Olive Oil
GAEs	Gallic Acid Equivalents
HAT	Hydrogen Atom Transfer
MCC	Mediterranean Culinary Center
MD	Mediterranean Diet
ORAC	Oxygen Radical Absorbance Capacity
TAC	Total Antioxidant Capacity
TE	Trolox Equivalents
TEAC	Trolox Equivalent Antioxidant Capacity
TP	Total Polyphenols
Trolox	6-hydroxy-2,5,7,8-tetramethylchroman-2-carboxylic acid
