# Supplementary material for: Antioxidant Capacity and Polyphenolic Profile of Extractable and Non-Extractable Fractions of Traditional Mediterranean Diet Recipes from Different Regions
Source: Antioxidants (Basel). 2026 Mar 18;15(3):377. doi: 10.3390/antiox15030377 (PMC13023574; doi:10.3390/antiox15030377)
Supplement: Supplementary file 1 [file antioxidants-15-00377-s001.zip › antioxidants-4168478-supplementary.pdf]

Antioxidant Capacity and Polyphenolic Profile of Extractable and Non-Extractable Fractions of Traditional Mediterranean Diet Recipes from Different Regions

Marta Cuenca-Ortola 1,2 , Monica Gandia 2 , Salah Chaji 3, Fatima Zahrae El Mossaid 3 , Said Ennahli 3, El Amine Ajal 4,*, Stefania Filice 5, Achraf Ammar 6,7 , Amparo Gamero 2,* and Antonio Cilla 1

[1] Nutrition and Food Science Area, Faculty of Pharmacy and Food Sciences, University of Valencia, Burjassot, 46100 Valencia, Spain; marta.cuenca@uv.es (M.C.-O.); antonio.cilla@uv.es (A.C.)

[2] Food Technology Area, Faculty of Pharmacy and Food Sciences, University of Valencia, Burjassot, 46100 Valencia, Spain; monica.gandia@uv.es

[3] Agro-pôle Olivier, National School of Agriculture, Meknès 50001, Morocco; salahchaji1@gmail.com (S.C.); fatimazahrae.elmossaid9@gmail.com (F.Z.E.M.); ennahlisaid@gmail.com (S.E.)

[4] UPR of Pharmacognosy, Faculty of Medicine and Pharmacy of Rabat, Mohammed V University, Rabat 6203, Morocco

[5] Microtarians SIS, Societe d'Impact Societal, Luxembourg 1746, Luxembourg; stefania.filice@microtarians.com

[6] Department of Training and Movement Science, Institute of Sport Science, Johannes Gutenberg University Mainz, 55128 Mainz, Germany; acammar@uni-mainz.de

[7] Research Laboratory, Molecular Bases of Human Pathology, Faculty of Medicine, University of Sfax, Sfax 30000, Tunisia

* Correspondence: e.ajal@um5r.ac.ma (E.A.A.); amparo.gamero@uv.es (A.G.)

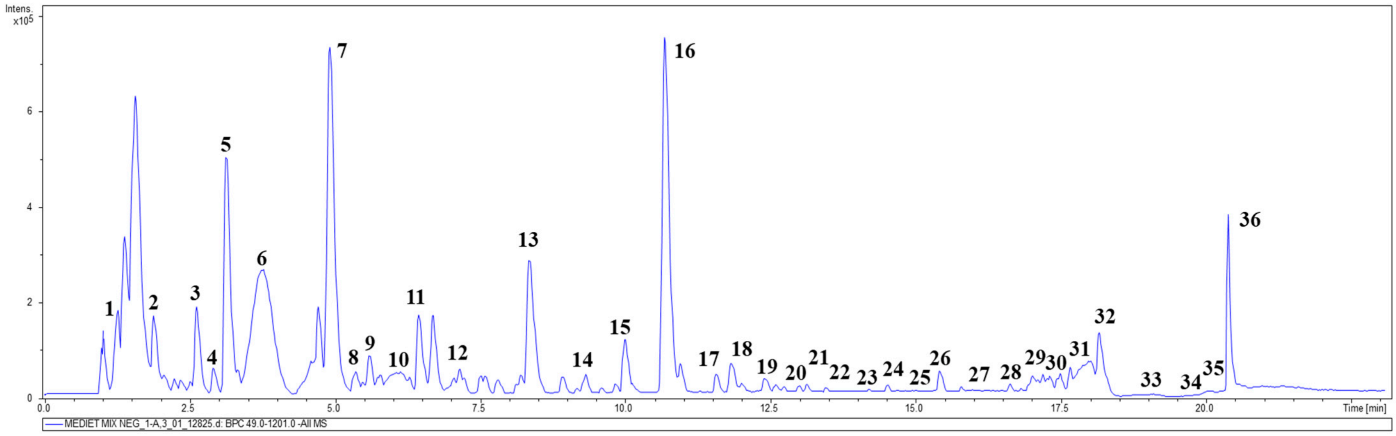

Figure S1. LC–MS base peak chromatogram (BPC) of the composite extract used for compound identification. Peaks are numbered according to their elution order and correspond to the compounds listed in Table S1.

Table S1. LC–MS characterization of the detected compounds, including retention time (RT), molecular formula, experimental m/z, mass error and mSigma values.

Pic number	Phenolic compounds	Chemical formula	Phenolic family	RT (min)	m/z	Error (mDa)	mSigma
1	**Citric acid**	$C_6H_8O_7$	Organic acid	1.25	191.0191	-0.651	3
2	**Hydroxytyrosol**	$C_8H_{10}O_3$	Simple phenol	2.32	153.0551	-0.594	0.2
3	**Protocatechuic acid**	$C_7H_6O_4$	Phenolic acid	2.63	153.019	-0.309	8.2
4	**Tyrosol**	$C_8H_{10}O_2$	Simple phenol	2.82	137.0608	-0.006	6.2
5	**Gallic acid**	$C_7H_6O_5$	Phenolic acid	3.15	169.014	-0.793	26.6
6	**Vanillic acid**	$C_8H_8O_4$	Phenolic acid	4.05	167.0345	-0.459	7.1
7	**Syringic acid**	$C_9H_{10}O_5$	Phenolic acid	4.40	197.0452	-0.004	12.4
8	**Chlorogenic acid**	$C_{16}H_{18}O_9$	Phenolic acid	5.12	353.0878	-0.221	10.8
9	**Caffeic acid**	$C_9H_8O_4$	Phenolic acid	5.75	179.0347	-0.31	8.3
10	**p-Coumaric acid**	$C_9H_8O_3$	Phenolic acid	6.27	163.0397	-0.107	11.8
11	**Ferulic acid**	$C_{10}H_{10}O_4$	Phenolic acid	6.81	193.0503	-0.57	7.6
12	**Sinapic acid**	$C_{11}H_{12}O_5$	Phenolic acid	7.33	223.0615	-0.045	9.9
13	**Rosmarinic acid**	$C_{18}H_{16}O_8$	Phenolic acid	8.92	359.0772	-0.186	11.5
14	**Catechin**	$C_{15}H_{14}O_6$	Flavan-3-ol	9.42	289.0718	-0.142	9.4
15	**Epicatechin**	$C_{15}H_{14}O_6$	Flavan-3-ol	9.85	289.0718	-0.118	9.8
16	**Procyanidin B1**	$C_{30}H_{26}O_{12}$	Condensed tannin	11.22	577.1351	-0.265	15.7
17	**Naringenin**	$C_{15}H_{12}O_5$	Flavanone	11.71	271.0613	0.086	13.4
18	**Apigenin-7-O-glucoside**	$C_{21}H_{20}O_{10}$	Flavone glycoside	12.12	431.099	0.667	26.9
19	**Quercetin-3-glucoside**	$C_{21}H_{20}O_{12}$	Flavonol glycoside	12.53	463.0882	-0.01	29.3
20	**Rutin**	$C_{27}H_{30}O_{16}$	Flavonol glycoside	13.15	609.1464	0.278	5
21	**Myricetin**	$C_{15}H_{10}O_8$	Flavonol	13.76	317.0303	-0.233	16.8
22	**Quercetin**	$C_{15}H_{10}O_7$	Flavonol	14.22	301.0351	-0.194	17
23	**Isorhamnetin**	$C_{16}H_{12}O_7$	Flavonol	14.46	315.051	-0.156	14.9
24	**Kaempferol**	$C_{15}H_{10}O_6$	Flavonol	14.55	285.0405	0.121	12.7
25	**Apigenin**	$C_{15}H_{10}O_5$	Flavone	14.95	269.0459	0.329	13.3
26	**Luteolin**	$C_{15}H_{10}O_6$	Flavone	15.15	285.0405	0.136	2.4
27	**Cyanidin-3-glucoside**	$C_{21}H_{21}O_{11}$	Anthocyanin	16.25	449.1095	0.53	3.4
28	**Cyanidin-3-rutinoside**	$C_{27}H_{31}O_{15}$	Anthocyanin	16.66	595.1657	0.412	5.8
29	**Delphinidin-3-rutinoside**	$C_{27}H_{31}O_{16}$	Anthocyanin	16.95	611.1607	0.468	6.6
30	**Pelargonidin-3-glucoside**	$C_{21}H_{21}O_{10}$	Anthocyanin	17.45	433.1129	0.387	4.9
31	**Verbascoside**	$C_{29}H_{36}O_{15}$	Phenylpropanoid glycoside	17.85	623.1981	-0.003	14.6
32	**Resveratrol**	$C_{14}H_{12}O_3$	Stilbene	18.85	227.0714	0.094	10.7
33	**Oleuropein**	$C_{25}H_{32}O_{13}$	Secoiridoid	19.15	539.1769	-0.116	11.1
34	**Pinoresinol**	$C_{20}H_{22}O_6$	Lignan	20.02	357.1337	-0.625	20.7
35	**Oleacein**	$C_{17}H_{20}O_6$	Secoiridoid	20.60	319.1187	-0.204	12.9
36	**Oleocanthal**	$C_{17}H_{20}O_5$	Secoiridoid	20.55	303.1238	-0.173	12.1

Table S2. Average contents (expressed as in mg/kg of DM) of individual phenolic compounds in recipes belonging to the Mediterranean European countries.

Phenolic compounds	Chemical formula	Phenolic family	RT (min)	F1	F2	F3	F4	F5	F6	F7	F8	I1	I2	I3	I4	I5	I6	I7	I8	S1	S2	S3	S4	S5	S6	S7	S8
**Citric acid**	$C_6H_8O_7$	Organic acid	**1.25**	262.92	7.13	ND	ND	17.01	ND	24.52	ND	117.83	ND	ND	ND	95.88	ND	89.73	ND	ND	ND	ND	ND	ND	ND	ND	ND
**Hydroxytyrosol**	$C_8H_{10}O_3$	Simple phenol	**2.32**	2.81	0.57	ND	1.57	7.29	ND	1.03	ND	21.45	4.03	178.21	12.00	24.97	ND	0.15	ND	12.68	19.98	1.23	0.54	0.73	0.69	0.54	3.85
**Protocatechuic acid**	$C_7H_6O_4$	Phenolic acid	**2.63**	ND	ND	ND	ND	ND	ND	ND	1.34	ND	ND	ND	ND	ND	ND	ND	0.80	ND	ND	ND	ND	ND	ND	ND	1.21
**Tyrosol**	$C_8H_{10}O_2$	Simple phenol	**2.82**	2.53	0.52	ND	1.42	2.73	ND	0.93	ND	15.73	3.63	128.82	9.26	18.88	ND	0.13	ND	9.19	14.44	1.11	0.49	0.66	0.62	0.49	3.12
**Gallic acid**	$C_7H_6O_5$	Phenolic acid	**3.15**	ND	0.37	2.46	5.39	0.35	0.30	0.66	ND	4.42	3.58	0.67	0.30	0.86	47.33	ND	2.50	4.89	2.76	ND	ND	19.85	ND	ND	3.07
**Vanillic acid**	$C_8H_8O_4$	Phenolic acid	**4.05**	ND	1.61	ND	ND	0.13	ND	3.00	1.38	ND	0.42	ND	0.72	ND	3.28	4.07	1.12	2.15	2.75	ND	ND	ND	ND	3.32	1.72
**Syringic acid**	$C_9H_{10}O_5$	Phenolic acid	**4.40**	ND	1.58	ND	ND	0.13	ND	2.95	1.36	ND	0.42	ND	0.70	ND	3.22	4.00	0.60	2.11	2.70	ND	ND	ND	ND	ND	0.96
**Chlorogenic acid**	$C_{16}H_{18}O_9$	Phenolic acid	**5.12**	244.55	206.49	43.45	ND	174.97	1.40	20.43	ND	98.20	ND	ND	ND	212.32	ND	74.78	221.80	40.17	ND	310.11	67.47	44.96	198.62	7.65	0.95
**Caffeic acid**	$C_9H_8O_4$	Phenolic acid	**5.75**	162.39	13.53	7.69	2.09	23.62	1.60	13.09	ND	61.41	0.55	0.28	0.35	60.37	ND	46.85	33.30	2.27	1.12	52.48	14.94	19.27	45.27	3.33	7.48
***p*-Coumaric acid**	$C_9H_8O_3$	Phenolic acid	**6.27**	6.54	3.58	0.29	1.26	0.51	0.91	13.38	5.00	0.03	4.83	0.15	2.73	4.80	21.34	14.73	11.10	8.19	10.47	11.93	4.46	7.63	6.03	10.75	6.32
**Ferulic acid**	$C_{10}H_{10}O_4$	Phenolic acid	**6.81**	0.32	89.72	0.24	0.50	7.15	0.35	341.71	120.20	0.01	139.16	0.04	62.24	ND	300.60	354.33	12.30	187.40	239.31	21.47	6.96	10.21	19.67	46.73	14.25
**Sinapic acid**	$C_{11}H_{12}O_5$	Phenolic acid	**7.33**	ND	60.77	ND	ND	ND	ND	ND	ND	ND	ND	ND	ND	ND	ND	ND	ND	ND	ND	ND	ND	ND	ND	ND	ND
**Rosmarinic acid**	$C_{18}H_{16}O_8$	Phenolic acid	**8.92**	299.33	2.26	ND	16.78	10.79	10.06	1.36	ND	ND	ND	3.72	7.16	ND	ND	1.68	ND	ND	ND	ND	ND	ND	ND	19.15	ND
**Catechin**	$C_{15}H_{14}O_6$	Flavan-3-ol	**9.42**	ND	23.05	11.31	ND	27.31	ND	ND	4.69	2.83	ND	ND	ND	ND	7.75	ND	10.30	6.54	ND	ND	ND	9.40	ND	0.79	9.84
**Epicatechin**	$C_{15}H_{14}O_6$	Flavan-3-ol	**9.85**	ND	ND	6.70	ND	8.10	ND	ND	3.75	1.55	ND	ND	ND	ND	ND	ND	30.80	0.74	ND	ND	ND	8.36	ND	0.32	12.35
**Procyanidin B1**	$C_{30}H_{26}O_{12}$	Condensed tannin	**11.22**	ND	ND	ND	ND	ND	ND	ND	ND	ND	ND	ND	ND	ND	ND	ND	20.50	ND	ND	ND	ND	ND	ND	ND	15.42
**Naringenin**	$C_{15}H_{12}O_5$	Flavanone	**11.71**	31.77	0.86	ND	ND	2.06	ND	2.96	ND	14.24	ND	ND	ND	11.59	ND	10.84	ND	ND	ND	ND	8.24	27.30	ND	0.37	4.20
**Apigenin-7-*O*-glucoside**	$C_{21}H_{20}O_{10}$	Flavone glycoside	**12.12**	3.87	0.08	ND	0.22	1.07	0.22	0.02	ND	0.16	0.30	0.05	0.09	1.49	3.86	0.01	ND	ND	ND	ND	2.44	ND	ND	0.26	ND
**Quercetin-3-glucoside**	$C_{21}H_{20}O_{12}$	Flavonol glycoside	**12.53**	ND	0.42	0.66	17.17	0.96	1.52	0.40	1.30	2.81	0.42	0.64	2.54	1.04	0.05	ND	ND	0.73	0.88	1.15	1.05	ND	3.09	1.71	ND

Table S2. *(continue).*

Phenolic compounds	Chemical formula	Phenolic family	RT (min)	F1	F2	F3	F4	F5	F6	F7	F8	I1	I2	I3	I4	I5	I6	I7	I8	S1	S2	S3	S4	S5	S6	S7	S8
**Rutin**	$C_{27}H_{30}O_{16}$	**Flavonol glycoside**	**13.15**	115.03	7.55	4.82	ND	14.99	ND	10.73	ND	56.60	ND	4.32	2.13	41.95	ND	39.26	0.30	ND	ND	ND	ND	ND	ND	ND	0.88
**Myricetin**	$C_{15}H_{10}O_8$	**Flavonol**	**13.76**	7.23	9.03	3.44	ND	9.60	0.04	0.61	1.01	3.41	ND	ND	ND	6.49	0.94	2.22	ND	2.05	ND	9.16	2.01	3.54	5.88	0.37	ND
**Quercetin**	$C_{15}H_{10}O_7$	**Flavonol**	**14.22**	ND	ND	1.29	34.12	1.64	2.90	0.77	2.48	5.56	0.81	1.26	4.45	2.07	0.10	ND	0.20	1.33	1.70	2.21	2.05	ND	5.92	2.56	1.34
**Isorhamnetin**	$C_{16}H_{12}O_7$	**Flavonol**	**14.46**	ND	ND	ND	ND	ND	ND	0.02	ND	ND	ND	ND	ND	ND	ND	ND	ND	ND	ND	ND	ND	ND	ND	1.54	ND
**Kaempferol**	$C_{15}H_{10}O_6$	**Flavonol**	**14.55**	ND	4.17	0.17	4.53	1.35	0.92	0.15	0.83	0.68	0.09	0.14	2.95	0.22	ND	ND	ND	0.57	0.23	0.30	0.19	ND	0.79	4.04	0.65
**Apigenin**	$C_{15}H_{10}O_5$	**Flavone**	**14.95**	3.69	0.07	ND	0.21	1.11	0.22	0.02	ND	0.15	0.29	0.05	0.09	1.47	3.62	ND	ND	ND	ND	ND	2.32	ND	ND	0.24	0.74
**Luteolin**	$C_{15}H_{10}O_6$	**Flavone**	**15.15**	2.98	0.05	ND	0.17	0.32	0.15	0.01	ND	0.11	0.22	0.04	0.07	0.95	3.15	0.03	ND	ND	ND	ND	1.99	ND	ND	0.19	0.69
**Cyanidin-3-glucoside**	$C_{21}H_{21}O_{11}$	**Anthocyanin**	**16.25**	ND	ND	ND	ND	ND	ND	ND	66.01	ND	ND	ND	ND	ND	ND	ND	ND	ND	ND	ND	ND	ND	ND	ND	ND
**Cyanidin-3-rutinoside**	$C_{27}H_{31}O_{15}$	**Anthocyanin**	**16.66**	ND	ND	ND	ND	ND	ND	ND	57.76	ND	ND	ND	ND	ND	ND	ND	ND	ND	ND	ND	ND	ND	ND	ND	ND
**Delphinidin-3-rutinoside**	$C_{27}H_{31}O_{16}$	**Anthocyanin**	**16.95**	9.77	2.05	0.44	ND	1.75	0.01	0.21	ND	0.97	ND	ND	ND	8.63	ND	0.74	ND	0.40	ND	3.15	0.68	0.45	1.95	0.08	ND
**Pelargonidin-3-glucoside**	$C_{21}H_{21}O_{10}$	**Anthocyanin**	**17.45**	0.47	0.01	ND	ND	0.03	ND	0.04	ND	0.22	ND	ND	ND	0.18	ND	0.17	ND	ND	ND	ND	0.13	0.41	ND	0.01	ND
**Verbascoside**	$C_{29}H_{36}O_{15}$	**Phenylpropanoid glycoside**	**17.85**	ND	ND	ND	ND	ND	ND	ND	ND	ND	ND	ND	ND	ND	ND	ND	ND	ND	ND	ND	ND	ND	ND	ND	ND
**Resveratrol**	$C_{14}H_{12}O_3$	**Stilbene**	**18.85**	ND	ND	ND	ND	ND	ND	ND	ND	0.03	ND	ND	ND	ND	ND	ND	ND	0.03	ND	ND	ND	ND	ND	ND	ND
**Oleuropein**	$C_{25}H_{32}O_{13}$	**Secoiridoid**	**19.15**	ND	ND	ND	ND	72.90	ND	ND	ND	39.72	ND	350.81	17.15	39.95	ND	ND	ND	24.71	39.44	ND	ND	ND	ND	ND	1.98
**Pinoresinol**	$C_{20}H_{22}O_6$	**Lignan**	**20.02**	0.71	0.15	ND	0.39	1.37	ND	0.26	ND	1.25	1.02	8.12	1.23	2.11	ND	0.04	ND	0.60	0.90	0.31	0.14	0.19	0.17	0.14	ND
**Oleacein**	$C_{17}H_{20}O_6$	**Secoiridoid**	**20.60**	53.37	10.89	ND	29.91	ND	ND	19.65	ND	30.19	76.64	53.32	65.07	94.88	ND	2.75	ND	6.26	5.00	23.32	10.23	13.93	13.01	10.24	2.41
**Oleocanthal**	$C_{17}H_{20}O_5$	**Secoiridoid**	**20.55**	61.80	12.61	ND	34.64	ND	ND	22.75	ND	34.95	88.74	61.74	75.34	109.87	ND	3.19	ND	7.25	5.79	27.01	11.85	16.13	15.07	11.86	2.97

Table S3. Average contents (expressed as in mg/kg of DM) of individual phenolic compounds in recipes belonging to the non-Mediterranean European countries.

Phenolic compounds	G1	G2	G3	G4	L1	L2	L3	L4
**Citric acid**	13.17	ND	ND	ND	ND	ND	ND	ND
**Hydroxytyrosol**	ND	ND	ND	ND	ND	ND	ND	ND
**Protocatechuic acid**	ND	ND	ND	2.05	ND	ND	ND	ND
**Tyrosol**	ND	ND	ND	ND	ND	ND	ND	ND
**Gallic acid**	38.40	ND	ND	4.67	30.24	ND	ND	ND
**Vanillic acid**	ND	1.30	ND	2.96	ND	ND	3.97	9.81
**Syringic acid**	ND	1.28	ND	2.51	ND	ND	ND	1.90
**Chlorogenic acid**	11.43	ND	261.10	1.48	ND	191.82	9.41	5.80
**Caffeic acid**	8.16	ND	26.35	3.53	21.25	36.51	2.33	2.50
***p*-Coumaric acid**	0.99	4.72	8.31	3.76	11.04	5.87	11.91	8.70
**Ferulic acid**	0.23	113.45	23.87	15.95	14.95	10.57	92.65	43.52
**Sinapic acid**	ND	ND	ND	ND	ND	ND	ND	ND
**Rosmarinic acid**	9.74	ND	4.97	ND	ND	ND	ND	ND
**Catechin**	18.24	ND	8.21	8.43	8.84	4.45	ND	1.58
**Epicatechin**	7.20	ND	6.16	10.25	ND	ND	ND	1.32
**Procyanidin B1**	ND	ND	ND	18.22	ND	ND	ND	ND
**Naringenin**	1.59	ND	ND	0.46	ND	ND	ND	ND
**Apigenin-7-*O*-glucoside**	0.20	ND	0.06	ND	1.92	ND	ND	ND
**Quercetin-3-glucoside**	3.54	8.32	0.98	ND	2.09	1.45	0.71	0.36
**Rutin**	5.76	49.13	ND	0.91	ND	ND	ND	ND
**Myricetin**	3.96	ND	9.58	0.04	1.05	6.21	0.33	0.52
**Quercetin**	6.97	16.40	1.89	1.82	3.96	2.50	1.40	0.71
**Isorhamnetin**	ND	ND	ND	ND	ND	ND	ND	ND
**Kaempferol**	0.72	1.87	0.25	0.46	1.32	2.01	ND	ND
**Apigenin**	0.20	ND	0.06	0.46	0.47	ND	ND	ND
**Luteolin**	0.13	ND	0.05	0.46	7.49	ND	ND	ND
**Cyanidin-3-glucoside**	ND	ND	ND	ND	ND	ND	ND	ND
**Cyanidin-3-rutinoside**	ND	ND	ND	ND	ND	ND	ND	ND
**Delphinidin-3-rutinoside**	0.45	ND	2.64	ND	ND	1.95	0.38	0.06
**Pelargonidin-3-glucoside**	0.02	ND	ND	ND	ND	ND	ND	ND
**Verbascoside**	ND	ND	ND	ND	ND	ND	ND	ND
**Resveratrol**	0.29	ND	ND	ND	ND	ND	ND	ND
**Oleuropein**	ND	ND	ND	ND	ND	ND	ND	ND
**Pinoresinol**	ND	ND	ND	ND	ND	ND	ND	ND
**Oleacein**	ND	ND	ND	ND	ND	ND	ND	ND
**Oleocanthal**	ND	ND	ND	ND	ND	ND	ND	ND

Table S4. Average contents (expressed as in mg/kg of DM) of individual phenolic compounds in recipes belonging to the Mediterranean African countries.

Phenolic compounds	A1	A2	A3	A4	A5	A6	A7	A8	M1	M2	M3	M4	M5	M6	M7	M8	T1	T2	T3	T4	T5	T6	T7	T8
**Citric acid**	ND	ND	ND	4.87	ND	ND	ND	44.88	ND	ND	ND	ND	ND	ND	ND	ND	ND	40.22	16.25	ND	35.09	9.81	19.03	ND
**Hydroxytyrosol**	0.16	0.19	1.01	0.81	74.79	1.70	ND	ND	4.36	2.73	1.12	15.13	4.12	1.81	1.30	4.41	0.73	0.50	0.85	0.46	3.05	0.34	0.37	ND
**Protocatechuic acid**	ND	ND	ND	ND	ND	ND	ND	ND	ND	ND	ND	ND	ND	ND	ND	1.36	ND	ND	ND	ND	ND	ND	ND	2.17
**Tyrosol**	0.15	0.17	0.91	0.73	37.39	1.53	ND	ND	3.92	2.46	1.01	7.88	3.71	1.63	1.17	3.52	0.65	0.45	0.76	0.41	2.74	0.31	0.33	ND
**Gallic acid**	7.91	30.27	60.69	92.35	ND	ND	ND	31.60	23.01	5.41	3.30	7.62	5.48	15.47	2.12	4.19	0.88	16.94	1.61	ND	3.29	2.91	0.79	6.24
**Vanillic acid**	5.59	3.04	ND	ND	ND	6.10	ND	ND	ND	0.20	2.76	ND	ND	ND	ND	1.95	3.27	ND	ND	ND	ND	ND	ND	1.95
**Syringic acid**	ND	ND	ND	ND	ND	ND	ND	ND	ND	ND	ND	ND	ND	ND	ND	1.07	ND	ND	ND	ND	ND	ND	ND	1.12
**Chlorogenic acid**	12.77	ND	ND	1.51	8.93	14.92	32.73	13.96	ND	10.47	ND	ND	46.77	63.47	20.56	ND	23.43	33.52	42.70	378.03	52.68	133.04	15.86	ND
**Caffeic acid**	2.46	0.98	1.31	1.68	2.59	4.07	6.22	15.08	14.70	3.33	0.17	1.97	15.22	17.65	5.43	8.21	6.55	21.37	22.12	75.07	24.24	63.22	11.27	7.86
***p*-Coumaric acid**	17.62	13.83	11.69	18.39	0.71	16.78	ND	7.75	8.76	4.67	7.17	1.08	3.73	1.85	0.25	5.08	12.34	13.48	4.68	15.92	2.04	14.31	0.75	6.38
**Ferulic acid**	94.72	67.77	17.87	28.33	1.03	91.53	0.36	5.17	11.57	21.88	53.24	0.72	5.49	1.48	0.14	12.31	49.32	19.49	5.51	31.84	3.92	32.06	0.38	15.42
**Sinapic acid**	ND	ND	ND	ND	ND	ND	ND	ND	ND	ND	ND	ND	ND	ND	ND	ND	ND	ND	ND	ND	ND	ND	ND	ND
**Rosmarinic acid**	ND	ND	ND	ND	ND	ND	ND	ND	ND	ND	ND	ND	ND	ND	ND	ND	ND	ND	ND	ND	ND	ND	ND	ND
**Catechin**	1.29	4.59	8.88	14.27	ND	1.70	21.65	ND	9.40	11.14	28.63	ND	12.55	ND	11.66	13.42	2.03	21.07	ND	ND	0.46	ND	ND	22.75
**Epicatechin**	ND	ND	ND	ND	ND	ND	15.64	ND	2.81	9.01	20.71	ND	10.04	ND	8.71	16.70	1.02	17.56	ND	ND	ND	ND	ND	28.63
**Procyanidin B1**	ND	ND	ND	ND	ND	ND	ND	ND	ND	2.95	ND	ND	ND	ND	ND	20.58	ND	10.54	ND	ND	ND	ND	ND	32.14
**Naringenin**	ND	ND	ND	0.92	ND	ND	ND	8.48	ND	1.21	ND	ND	ND	ND	ND	ND	4.81	4.86	19.66	7.02	4.24	1.36	2.30	ND
**Apigenin-7-*O*-glucoside**	0.62	2.22	4.43	7.15	1.03	ND	ND	ND	0.94	0.60	ND	0.34	5.13	0.08	0.48	ND	ND	0.20	ND	ND	0.22	0.93	ND	ND
**Quercetin-3-glucoside**	0.14	0.40	0.37	1.59	0.56	ND	3.89	4.95	ND	0.55	3.81	6.96	0.37	4.85	4.26	ND	2.92	0.60	0.42	ND	0.67	16.17	ND	ND
**Rutin**	ND	ND	ND	0.24	ND	ND	ND	ND	ND	ND	ND	ND	ND	14.10	ND	ND	ND	17.60	7.30	ND	15.75	49.82	8.33	ND
**Myricetin**	0.54	0.54	1.07	1.76	0.27	0.64	6.19	0.43	1.44	3.04	6.77	ND	4.11	1.89	3.57	ND	1.08	7.03	1.27	11.26	1.62	4.00	0.48	ND
**Quercetin**	0.27	0.78	0.72	3.08	1.11	ND	7.91	9.57	ND	1.09	7.57	13.30	0.73	9.63	8.62	27.38	5.15	1.15	0.81	ND	1.31	31.88	ND	4.72
**Isorhamnetin**	ND	ND	ND	ND	ND	ND	3.11	ND	ND	ND	1.91	0.07	0.02	0.20	2.27	1.12	1.24	ND	ND	ND	ND	ND	ND	ND
**Kaempferol**	0.04	0.07	0.08	0.71	0.08	ND	0.03	1.20	ND	0.12	0.16	1.83	0.10	0.42	0.33	2.61	3.66	0.27	0.17	ND	0.27	1.10	ND	1.07

Table S4. *(continue).*

Phenolic compounds	A1	A2	A3	A4	A5	A6	A7	A8	M1	M2	M3	M4	M5	M6	M7	M8	T1	T2	T3	T4	T5	T6	T7	T8
**Apigenin**	0.58	2.04	4.15	6.67	0.96	ND	ND	ND	ND	0.54	ND	0.30	3.93	0.08	0.42	2.76	ND	0.18	ND	ND	0.21	0.87	ND	0.85
**Luteolin**	0.50	1.79	3.61	5.84	0.75	ND	ND	ND	4.71	0.57	ND	0.36	7.71	0.08	0.52	3.17	ND	0.16	ND	ND	0.18	0.74	ND	0.92
**Cyanidin-3-glucoside**	ND	ND	ND	ND	ND	ND	ND	ND	ND	ND	ND	ND	ND	ND	ND	ND	ND	ND	ND	ND	ND	ND	ND	ND
**Cyanidin-3-rutinoside**	ND	ND	ND	ND	ND	ND	ND	ND	ND	ND	ND	ND	ND	ND	ND	ND	ND	ND	ND	ND	ND	ND	ND	ND
**Delphinidin-3-rutinoside**	0.13	ND	ND	0.02	0.09	0.15	0.33	0.14	ND	0.11	ND	ND	0.47	0.63	0.21	ND	0.24	0.33	0.43	3.82	0.52	1.31	0.16	ND
**Pelargonidin-3-glucoside**	ND	ND	ND	0.01	ND	ND	ND	0.13	ND	0.02	ND	ND	ND	ND	ND	ND	0.07	0.07	0.30	0.11	0.06	0.02	0.03	ND
**Verbascoside**	ND	ND	ND	ND	17.36	ND	ND	ND	ND	ND	ND	ND	ND	ND	ND	ND	ND	ND	ND	ND	ND	ND	ND	ND
**Resveratrol**	ND	ND	ND	ND	ND	ND	ND	ND	ND	ND	ND	ND	ND	ND	ND	ND	ND	ND	ND	ND	ND	ND	ND	ND
**Oleuropein**	ND	ND	ND	ND	253.74	ND	ND	ND	ND	ND	ND	37.63	ND	ND	ND	2.43	ND	ND	ND	ND	ND	ND	ND	ND
**Pinoresinol**	0.04	0.05	0.26	0.21	5.04	0.44	ND	ND	1.09	0.69	0.29	1.76	1.04	0.47	0.33	ND	0.18	0.12	0.22	0.12	0.77	0.09	0.09	ND
**Oleacein**	3.10	3.67	19.18	15.41	ND	32.20	ND	ND	82.85	51.87	21.27	76.16	78.30	34.36	24.76	2.94	13.78	9.48	16.08	8.72	57.90	6.47	6.97	ND
**Oleocanthal**	3.59	4.25	22.21	17.84	ND	37.29	ND	ND	95.93	60.06	24.63	88.19	90.66	39.78	28.67	3.71	15.96	10.97	18.62	10.09	67.04	7.49	8.08	ND
